# Rare variants in the endocytic pathway are associated with Alzheimer’s disease, its related phenotypes, and functional consequences

**DOI:** 10.1371/journal.pgen.1009772

**Published:** 2021-09-13

**Authors:** Lingyu Zhan, Jiajin Li, Brandon Jew, Jae Hoon Sul

**Affiliations:** 1 Molecular Biology Institute, David Geffen School of Medicine, University of California, Los Angeles, Los Angeles, California, United States of America; 2 Department of Human Genetics, David Geffen School of Medicine, University of California, Los Angeles, Los Angeles, California, United States of America; 3 Interdepartmental Program in Bioinformatics, University of California, Los Angeles, Los Angeles, California, United States of America; 4 Department of Psychiatry and Biobehavioral Sciences, University of California, Los Angeles, Los Angeles, California, United States of America; University of Miami, Miller School of Medicine, UNITED STATES

## Abstract

Late-onset Alzheimer’s disease (LOAD) is the most common type of dementia causing irreversible brain damage to the elderly and presents a major public health challenge. Clinical research and genome-wide association studies have suggested a potential contribution of the endocytic pathway to AD, with an emphasis on common loci. However, the contribution of rare variants in this pathway to AD has not been thoroughly investigated. In this study, we focused on the effect of rare variants on AD by first applying a rare-variant gene-set burden analysis using genes in the endocytic pathway on over 3,000 individuals with European ancestry from three large whole-genome sequencing (WGS) studies. We identified significant associations of rare-variant burden within the endocytic pathway with AD, which were successfully replicated in independent datasets. We further demonstrated that this endocytic rare-variant enrichment is associated with neurofibrillary tangles (NFTs) and age-related phenotypes, increasing the risk of obtaining severer brain damage, earlier age-at-onset, and earlier age-of-death. Next, by aggregating rare variants within each gene, we sought to identify single endocytic genes associated with AD and NFTs. Careful examination using NFTs revealed one significantly associated gene, *ANKRD13D*. To identify functional associations, we integrated bulk RNA-Seq data from over 600 brain tissues and found two endocytic expression genes (eGenes), *HLA-A* and *SLC26A7*, that displayed significant influences on their gene expressions. Differential expressions between AD patients and controls of these three identified genes were further examined by incorporating scRNA-Seq data from 48 post-mortem brain samples and demonstrated distinct expression patterns across cell types. Taken together, our results demonstrated strong rare-variant effect in the endocytic pathway on AD risk and progression and functional effect of gene expression alteration in both bulk and single-cell resolution, which may bring more insight and serve as valuable resources for future AD genetic studies, clinical research, and therapeutic targeting.

## Author summary

Late-onset Alzheimer’s disease (LOAD) is the most common type of dementia and a leading cause of death in the world. Clinical and genetic studies have suggested the potential contribution of the cellular transportation pathway to AD with an emphasis on common variants. In this study, we investigated the effect of rare variants within the cellular transportation pathway and examined three large datasets with over 3,000 individuals with European ancestry. We reported enrichment of rare deleterious variants in the cellular transportation pathway in AD patients from all three datasets. We also observed an elevation of rare deleterious variants in this pathway was associated with individuals with severer brain damages (AD progression), earlier age-at-onset, and earlier age-of-death. By aggregating rare variants in each gene from the cellular transportation pathway, we revealed one gene in which rare variants were significantly associated with the progression of AD. By integrating gene expression data from brain tissues, we identified two additional genes whose rare-variant effect displayed significant influences on gene expression. Taken together, our results demonstrated that rare-variant effect in the cellular transportation pathway is strongly associated with the risk and the progression of AD, which may serve as future clinical and therapeutic targets.

## Introduction

Alzheimer’s disease (AD) is a destructive and irreversible neurodegenerative disorder, predominantly targeting the elderly.[1] It accounts for 60–70% of dementia cases, characteristic of progressive disintegration of cognitive functions, language ability, and memory loss.[1,2] Late-onset Alzheimer’s Disease (LOAD) is a subcategory of AD that appears in persons aged 65 years or older, showing a greater incidence rate as age increases.[3] As the population of Americans age 65 and beyond is expected to reach 88 million by 2050, the number of new AD cases is predicted to double and the prevalence rate to quadruple [4,5].

AD is known to have a substantial genetic component with multiple modulating genes. One of the strongest risk factors for LOAD is *APOE*. Recent GWASs have identified over 50 risk loci accounting for, together with all common SNPs, over 33% of the overall estimated heritability [6–12] that cohered into three major AD-related biological pathways: the cholesterol metabolism pathway, the immune response pathway, and the endocytic pathway.[13] While AD studies have mostly focused on the effect of common variants, such as in the lipid metabolism and immune system/response pathways implicated in recent GWASes, rare variants in genes related to these pathways have not yet been thoroughly investigated.[11,12,14–20] Among these implicated pathways, the endocytic pathway has been identified as one of the most prominent targets, where the earliest morphological changes can be observed as endosome enlargement in post-mortem brains from sporadic AD patients, as well as in some familial cases.[21,22] This phenomenon can be viewed as nearly diagnostic precision and served as blood-cellular markers.[23,24] These findings have also been supported by a recent genetic study showing the enrichment in clathrin-mediated/early endocytosis [25] and clinical research on the facilitation of Aβ clearance by LC3-associated endocytosis.[26] Previous studies using common variants have also identified several risk loci in the endocytic pathway, including *BIN1*, *PICALM*, *CD2AP*, *EPHA1*, and *SORL1* [27].

However, despite being one of the histological hallmarks of AD, few studies have examined the effect of rare variants within this endocytic pathway on AD pathogenic progression.[13] It is thus of interest to study the rare-variant effect on AD in this pathway. One major challenge in the rare variant study is the lack of power due to their rarity. In this study, to overcome this issue, we analyzed large-scale whole-genome sequencing (WGS) datasets that were recently developed for the study of AD-related traits, including the Alzheimer’s Disease Sequencing Project (ADSP) and the Accelerating Medicines Partnership-Alzheimer’s Disease (AMP-AD). Another efficient tool we leveraged to increase the power was a gene-set burden analysis, where we focused on the collective rare variant effect within a set of genes of a known biological pathway, rather than the effect of single variants or single genes, and thus avoided the multiple testing burden required otherwise. This method has helped identify risk genes in various complex traits, such as in central nervous system pathways of schizophrenia.[28–39] In some studies, this method has led to the discovery of novel biological pathways and therapeutic targets through the identification of gene networks participating in the same functional processes.[40–45] Similar gene-set analyses focusing on biological pathways, as well as gene-ontology-based pathway/module analyses, have also been effectively demonstrated in AD studies [11,46,47].

Therefore, in the current study, we included three large-scale WGS datasets with a total of 3,255 individuals of European ancestry, meta-analyzed under a gene-set rare-variant burden analysis framework. Phase 1 of this framework aimed to explore the effect of rare variants in the endocytic pathway as a whole and consisted of two stages followed by meta-analysis. Besides AD status, we additionally explored three AD-related phenotypes, neurofibrillary tangles (NFTs), age-at-onset (AAO), and age-of-death (AOD), along with the phase 1 analysis. NFT status was measured as Braak stages, first proposed by Braak and Braak in 1991, and served as a histopathological indicator of AD, [48–50] representing a finer progression of AD. Phase 2 of this framework was to identify single endocytic genes driving the rare-variant association we captured in phase 1. For each dataset, we examined each gene in the endocytic pathway using both AD and NFT status, followed by meta-analysis across all datasets. Finally, in phase 3, we sought to explore the functional consequences of the rare-variant effect identified in previous phases by examining both the bulk and single-cell expression of endocytic genes in relationship with AD status.

## Methods

### Study sample

To identify AD-associated rare-variant effects, we evaluated three publicly available large-scale WGS datasets collected for LOAD patients, downloaded as multi-sample VCF files. The Alzheimer’s disease sequencing project (ADSP) Umbrella is a collection of sequencing data from the ADSP and other AD and Related Dementia studies. Under this Umbrella, the ADSP group sequenced a large number of well-characterized Alzheimer’s Disease (AD) patients at three National Human Genome Research Institute Genome Centers (NHGRI) (Baylor College of Medicine Human Genome Sequencing Center, the Broad Institute, and the McDonnell Genome Institute at Washington University). The ascertainment methods and inclusion criteria are described in detail on the National Institute on Aging Genetics of Alzheimer’s Disease Data Storage Site (NIAGADS).[51,52] The sequencing results were mapped to the human reference genome (GRCh38) and processed using the VCPA 1.0 pipeline, which follows GATK best-practices pipeline.[53] Details of the variant calling pipeline can also be found on the NIAGADS. The ADSP discovery extension phase sequenced whole genomes of 1,466 cases and 1,534 controls from five cohorts provided by the Alzheimer’s Disease Genetics Consortium (ADGC) and included samples with diverse ancestry backgrounds (Non-Hispanic White, Caribbean Hispanic, and African American). Another WGS project shared under the ADSP Umbrella is the Alzheimer’s Disease Neuroimaging Initiative (ADNI), which is a longitudinal multi-center (63 sites across North America) study designed for early detection and tracking of AD. The ADNI WGS data contains 808 participants with 238 AD cases, 322 mild cognitive control (MCI) subjects, and 248 controls. A full list of the ascertainment methods and inclusion criteria can be found in detailed descriptions in the online ADNI protocol.[54] As of 2018, the ADNI was recalled under the same VCPA 1.0 pipeline as the ADSP discovery extension WGS data and mapped to the same human reference genome (GRCh38), which were then released together. This combined ADSP case-control dataset contained WGS data from a total of 3,896 individuals (accessed by us on Nov 20, 2018), which then underwent a sequence of quality control steps discussed later before including in our stage 1 analysis. Detailed demographic information of this dataset can be found in Table 1 and the distribution of age among AD cases and controls in S12 Fig. To note, we removed samples in the MCI category to ensure a strict bipartite definition of disease status from all our analyses.

**Table 1:** Summary of clinical, demographic, and technical information of individuals from three large WGS datasets.

WGS Datasets	Case-control		Family
Studies	ADSP	AMP-AD	ADSP
Total sample size	1,291	1,611	353
EUR Population (%)	41%	93%	50%
AD Patients	664	642	209
Controls	627	969	144
Males (%)	53.4%	35.4%	65.7%
APOE *ε*4 carriers (%)	43.5%	38.5%	44.8%
Reference genome	GRCh38	GRCh37	GRCh38

The numbers were counted only among the samples included in this current study. The percentages of EUR population were based on the total number of samples within each dataset and only the samples with EUR ancestry were included in this study, which served as the total input sample size in the first row. Abbreviations: AD: Alzheimer’s disease; WGS: whole-genome sequencing; EUR: European; ADSP: the Alzheimer’s disease sequencing project; AMP-AD: the Accelerating Medicines Partnership-Alzheimer’s Disease.

Our stage 2 replication included 1,894 WGS samples from the Accelerating Medicines Partnership-Alzheimer’s Disease (AMP-AD) Target Discovery and Preclinical Validation Project (accessed by us on Dec 13, 2018). The samples were separately sequenced at three centers: the Religious Orders Study and Memory and Aging Project (ROSMAP) (1,200 samples), the Mount Sinai Brain Bank (MSBB) study (354 samples), and the Mayo Clinic Brain Bank (Mayo) (350 samples). Previously reports have the detailed data collection scheme and sample inclusion and exclusion criteria.[55–58] This sequence data were mapped to the human reference genome (GRCh37) and were processed using the GATK best-practices workflow v3.4.0.[58] Another stage 2 replication was performed on the ADSP discovery extension phase family samples, which were released together with the ADSP case-control data. Therefore, this family WGS data were also mapped to the human reference genome (GRCh38) and processed using the VCPA 1.0 pipeline. The ADSP WGS family dataset contains 888 samples from 161 multiplex families. The inclusion criteria prioritized families loaded with LOAD with minimal *APOE* ε4 alleles. A detailed description of the study design and sample ascertainment methods can be found in previous reports.[59,60] For AMP-AD case-control study, this resulted in 642 AD patients and 969 controls after removing low-quality samples. For the ADSP family study, we obtained 545 AD patients and 285 cognitively normal older individuals (Table 1).

RNA-Seq data used for functional analysis were also obtained from the ROSMAP study of the AMP-AD Consortium [58]. The bulk RNA-Seq data were generated for 636 samples (254 AD cases, 368 controls, 12 other dementia, and two without annotation) from the dorsolateral prefrontal cortex (DLPFC) tissues by the Broad Institue’s Genomics Platform and processed in an automatic and parallelized pipeline.[55] The ROSMAP group also selected 48 post-mortem samples (24 with severe AD pathology and 24 with low-to-no pathology) and conducted droplet-based single-nucleus RNA sequencing of the prefrontal cortex region.[61] Metadata of the RNA-Seq data were then used to map samples to cases and controls following the same rule as in stage 2 replication, as well as to merge with genotyping data.

### Data processing and quality control of WGS data

#### Individual-level quality control of WGS data

We conducted stringent quality control (QC) to ensure that we only include high-quality samples. As the X chromosome was not available in the ADSP datasets, we did not include X chromosome for all analyses. Before checking the sequencing quality of each individual, we first removed variants failing Variant Quality Score Recalibration (VQSR) in the GATK pipeline and set all variants with genotyping quality (GQ) below 21 to missing. We included only bi-allelic variants for all future analyses. Within the remaining variants, for each individual, we evaluated the genotype missing rate, calculated theoretical relatedness to check for unexpected relationships by study design, and performed principal component analysis (PCA) to identify ancestral composition and population outliers. For the individual-level missing rate QC, we set the cutoff at 5% and removed all individuals beyond this threshold. For the relatedness check, we used PLink 1.9[62] and conducted identity by descent (IBD) analysis, which allowed us to compute a relatedness degree for each sample. For case-control studies, we retained only one in each cluster of samples estimated to be first- or second-degree relatives or duplicates within the corresponding cluster. For the ADSP family study, we compared the empirical kinship relationship record to our computed theoretical relatedness. For PCA, we used 1000 Genomes (1KG) phase 3 as a reference panel.[63] We used EIGENSTRAT [64] for PCA and included only independent common SNVs that were shared between 1KG and our dataset. To note, as PCA assumes unrelated individuals, when performing PCA for the ADSP family cohort, we restricted to only one sample in each family to avoid confounding ancestral relationship by kinship relationship. After having determined the ancestry of the included sample based on PCA, we then assigned that ancestry to the entire family of the included sample. PCA plots (PC1 vs. PC2) of all three datasets could be found in S4 and S6 Figs. As the X-chromosome was available for the AMP-AD study, we also performed sex-check for the AMD-AD and obsevered no sex-mismatched samples. In summary, after stringent sample-level quality control and the careful examination of ancestral backgrounds, we identified 1,291 (664 AD cases and 627 cognitively normal older controls), 1,611 (642 AD cases and 969 controls), and 353 (144 AD cases and 209 controls) high-quality European samples in the ADSP case-control, the AMP-AD case-control, and the ADSP family datasets, respectively, which then served as the primary objects of our study in both stages 1 and 2.

#### Variant-level QC of WGS data

We conducted stringent variant-level quality control to ensure keeping only high-quality SNVs. We included only variants that served as inputs for the individual-level QC while including only samples passing the individual-level QC. For each variant, we assessed the genotype missing rate, computed minor allele frequency (MAF) using all European samples, and calculated the Hardy-Weinberg Equilibrium (HWE) p-values using only unaffected European samples. For the variant-level missing rate QC, we set the cutoff at 2% and removed all variants beyond this threshold. For HWE, we set the cutoff at 0.001 for rare variants and removed all rare variants falling the HWE check where rare variants are defined in the following section. The number of variants passing the HWE filter could be found in S14 Table.

### Identification and annotation of rare variants

To identify rare variants, we used both external and internal sources of allele frequency to avoid potential inflation of the allele frequency introduced by the study design. For the external sources, we looked at the Europeans (EUR) in 1KG[63] and Non-Finnish Europeans (NFE) in the gnomAD v2 database [65], which matched the ancestral backgrounds of our datasets. We used two different MAF thresholds (0.1% and 1%) to define rare variants, as there is no one consensus definition of rarity and we will correct for testing multiple MAF thresholds in future analysis. In practice, when a variant was present in either of 1KG EUR or gnomAD v2 NFE samples and below the aforementioned threshold, we would keep it for further analysis. For the internal sources, we retained only samples with European ancestry based on the previous PCA, as different ancestral groups would have different allele frequency distributions. Then when a variant was absent in both external databases, we would look at the MAF estimated from the European samples within our dataset and selected rare variants based on 0.1% and 1% MAF thresholds separately. We then annotated rare variants using Ensembl Variant Effect Predictor (VEP)[66]. We defined a variant to be ‘deleterious’ if it is within one of the following categories: stop-gain, stop-loss, frameshift, splice-donor, splice-acceptor, and missense variants. Particularly, for missense variants, we additionally consulted PolyPhen-2[67] and retained only confident missense variants predicted to be ‘damaging.’ This definition of deleteriousness focused on coding regions, primarily due to the fact that the effect of non-coding variants was challenging to predict.[68,69] A distribution of variant types and singletons among the selected set of rare deleterious variants could be found in S9 Fig and S11 Table. In an additional validation of the deleteriousness, we further introduced the CADD score [70] as a third deleterious criterion in phase 1 analysis combined with VEP and PolyPhen-2. The distribution of CADD scores among the set of rare deleterious variants could be found in S8 Fig. As suggested by the CADD documentation, variants with scaled CADD > 15 were retained as pathogenic variants and the set of rare deleterious/pathogenic variants passing all three annotation tools were used in this validation test.

### Identification of genes in endocytic pathways

We identified genes involved in endocytic pathways using AmiGO 2 [71,72] gene ontology database to select all genes participating in this pathway. We identified three specific GO terms related to the endocytic system in the Homo Sapiens category, which corresponded to three specific compartments in the endocytic system (endosome, lysosome, and trans-Golgi network). The endosome compartment is a membrane-bound vacuole in eukaryotic, participating in the endocytic trafficking from the trans-Golgi network to the plasma membrane and vice versa.[73] The trans-Golgi network serves as an interconnected tubular network and the final cisternal structure involved in packaging and transporting of cargos to the lysosome, endosome, and cell surface.[74] The lysosome, a small membrane-bound lytic vacuole, is one of the end-point in the endocytic transporting pathway, which contains hydrolytic enzymes to break down various biomolecules.[75] The combination of these three compartments formed the essential backbone of the endocytic system, which we named as “endo-system” and used this term throughout the paper. After removing duplicates, we obtained 1,435 genes in total in the endo-system, while the three compartmental gene-sets contained 899 (endosome), 678 (lysosome), and 236 (trans-Golgi network) genes, respectively (S16 Table). We confirmed their biological functions with a functional enrichment analysis using the Database for Annotation, Visualization, and Integrated Discovery (DAVID)[76], where the top enriched GO terms were indeed lysosome, endosome, and trans-Golgi network. (S13 Fig) A comparison of the endo-system gene-set to the findings in the recent AD GWASes [11,12] has been provided by checking the number of endocytic genes implicated in Jansen et al. and Kunkle et al. (S7 Fig). To note, some genes were related to multiple compartmental gene-sets and thus only one of the duplicated genes was included in the endo-system gene-set (S11 Fig).

### Analysis of association between the burden of rare deleterious SNVs and AD status

To identify whether rare variants in the endocytic pathway are associated with AD, we compared the burden of rare deleterious SNVs between AD patients and controls. The burden was defined as the fraction of the alternative minor alleles that each individual carried for all rare deleterious SNVs, using the—score function in PLINK [62]. We additionally performed this procedure on the three compartmental gene-sets and obtained a burden score for each individual within each gene-set. To correct for potential confounding factors, for each gene-set, we first regressed the burden against the total number of rare SNVs and the top ten principal components (PCs). Due to randomness, the distribution of the number of rare SNVs might be naturally variable from sample to sample, in which case the distribution of rare deleterious SNVs would also be greatly affected. Similarly, the PCs helped to correct for potential population stratification within European ancestries. Both aspects could influence the burden score in ways unrelated to AD and thus need to be controlled. Once we had removed the confounding covariates, we performed three logistic regression models as proposed by Zhang et al.[77] using the residuals and AD status for all case-control studies. The three models differed in the covariates they corrected for. The minimal adjustment Model 0 (M0) controlled for the ten PCs and sequencing centers. This model has been previously reported to improve power for detecting variants whose effects are confounded with age and sex.[60] This phenomenon could be introduced by study design where the mean age between cases and controls are substantially disproportionate, as in the case of ADSP studies. Model 1 (M1) was built upon M0 by additionally including age and sex. Model 2 (M2) was further built upon M1 and included the count of *APOE* ε2 and ε4 alleles. For the ADSP family dataset, we generated kinship matrices and used a generalized linear mixed model (GLMM) to take kin relationships into consideration when calculating association p-values. In particular, we used the glmmkin function in the R package, GMMAT.[78] We computed odds ratio (OR) and p-values of association between the burden of rare deleterious SNVs and AD status in each model for European samples in each dataset (ADSP case-control study, AMP-AD case-control study, and ADSP family study). Our stage 1 analysis involved only the ADSP case-control dataset as the discovery set, while the AMP-AD case-control and the ADSP family study served as replication sets in our stage 2 analysis. We chose this analysis scheme because the ADSP case-control study encompassed the largest sample size, including non-European samples, even though we identified fewer samples with European ancestry compared to the AMP-AD case-control study. To note, the AMP-AD case-control study provided only the age-of-death for each individual, while the ADSP case-control and family studies provided only the age-at-onset. As a result, we used different definitions of age in analyzing different datasets. To validate our gene-set AD association analysis, we tested two additional methods provided by MAGMA [79] using the same set of rare deleterious variants. The first was the SNP-wise method applicable to both common and rare variants and the second was the burden method that MAGMA suggested to use for rare-variant-only analysis and was similar to the aforementioned gene-set AD association analysis using PLINK. We applied both methods to the set of rare deleterious variants previously defined and computed two types of p-values: a competitive p-value that tests whether the association within the gene-set is greater than in other genes and a self-contained p-value that tests whether there is an association within the gene-set of interest at all. The latter concept is the same as what our main analysis method aimed for. Due to our study design with multiple gene-sets and MAF thresholds, a Bonferroni correction was applied in accordance with the number of tests we performed in each analysis to define the study-wide significance threshold in each stage and each dataset. Although our analysis started with the whole endocytic pathway and then moved onto individual compartments, we, nonetheless, utilized a stringent multiple-testing correction threshold. Specifically, as we tested for four gene-sets (endo-system gene-set and three sub-compartmental gene-sets) and two MAF thresholds (1% and 0.1%), we set our significant threshold at α = 0.05/8 = 0.00625 for both stage 1 discovery phase and stage 2 replication phase analyses. Accordingly, we set our nominal significance threshold at α = 0.05.

To combine results from two stages (three studies) for each of the four gene-sets we tested previously, we performed meta-analyses on p-values using estimates from our best model, namely the model producing the smallest p-values among the three models tested. We used two meta-analysis methods to combine the results. The first was a fixed-effects inverse variance weighted method in METAL [80], which took ORs, standard deviations (SDs), and p-values for separate tests and combined them into one ‘Gene-set level’ p-value with an estimate of the unified effect. The second was Fisher’s method which only required p-values and has been shown to be more robust to some situations where a small portion of p-values are very small.[81,82] In particular, we used the sumlog function from the R package, ‘metap,’[83] which took into account the direction of effects in each study and the corresponding p-values. It then computed a ‘Gene-set level’ p-value similar to METAL indicating the significance of rare variants’ effect shared across studies but without an estimated effect size.

### Analysis of association between the burden of rare deleterious SNVs and AD-related phenotypes

To test for association between the burden of rare deleterious SNVs and NFTs, we leveraged the Braak stages and followed a similar workflow as in testing AD status. As the sample size of patients with Braak staging information was limited in the ADSP family study, we tested for replication only in AMP-AD case-control study after analyzing the ADSP case-control study in stage 1. We obtained 626 and 1,399 individuals with Braak staging information in ADSP and AMP-AD case-control datasets, respectively. To note, even though the ADSP case-control study had fewer samples with Braak staging information, we, nonetheless, followed the same analysis scheme as in the previous AD analysis. In practice, after removing confounding effects from the burden score, we applied three ordinal logistic regression (OLR) models (M0, M1, M2) to account for multiple ordered categories present in the Braak staging (stage 0 to VI). The regular logistic regression only allows binary dependent variables, which is not feasible for Braak stages. In particular, we used the polr function from the R package, MASS [84], which fits a logistic regression model to an ordered factor response. Similar to the previous burden analysis, our M0 accounted for sequencing centers and the top 10 PCs; our M1 additionally controlled for sex and age; finally, our M2 further included the count of *APOE* ε2 and ε4 alleles. For analyses in all datasets, our significance threshold after the multiple-testing correction was still at α = 0.00625 because we tested for two MAF thresholds and four gene-sets. Finally, the nominal significance threshold was also at α = 0.05. To increase statistical strength and precision in estimating effects [85], we again performed meta-analyses and combined these two independent tests similar to what we did for AD association analyses.

We additionally tested the age-specific risk of rare deleterious SNVs in the endocytic pathway. As aforementioned, the AAO and AOD information was provided by the ADSP studies and the AMP-AD study, respectively, which allowed us to test for two different age-specific risks within each gene-set. Different from AD risk, age-specific risk leveraged the information of age and estimated the association between the age-to-event (survival time) of patients and the rare-variant burden score. Therefore, we adopted a genetic epidemiological framework proposed by Desiken et al.[86], in which a Cox Proportional Hazard Regression (CPHR) was performed to account for age-to-event information. Specifically, we first used the Surv function from the R package, “survival”[87], and computed a survival time for each sample in each dataset. Then, we conducted CPHR using the coxph function from the R package, ‘survminer’[88], to estimate the hazard ratio, or the ratio of risk-to-event (onset or death), depending on the input age we used. We performed three CPHR models (M0, M1, and M2) similar to the previous burden analysis on AD status and Braak staging, except that age was not a covariate in either of the three models. Therefore, since we tested for two different MAF thresholds and four gene-sets (though in a stepwise fashion), we set a stringent significant threshold at α = 0.05/8 = 0.00625 and our nominally significant threshold at α = 0.05 for analyses in all three datasets. Finally, we combined the results of AAO in the same way as we did for AD and NFT association tests. The resulting p-value then indicated the shared rare-variant effect on AAO-specific risk across the ADSP case-control and family studies.

### Single-gene analysis

To identify specific genes within the endocytic pathway associated with AD, we extracted rare deleterious SNVs as defined previously for each gene in the endo-system gene-set that were present in European samples for the ADSP case-control, the AMP-AD case-control, and the ADSP family study. Association test was performed for AD status by first building a null model using the SKAT_Null_Model function in the R package, SKAT, [89] followed by running the SKATBinary function using the SKAT-O feature to obtain association p-values for binary traits. We used a full model that included age, sex, sequencing center, the number of *APOE* ε2 and ε4 alleles, and top 10 PCs. To note, we also applied SKAT_Null_Model to the ADSP family dataset without incorporating kinship structure. This procedure could only be valid in the case where the family structure was relatively simple and did not contribute to a large effect in our analysis. By re-running the previous AD burden analysis with and without kinship information, we indeed observed only small deviations between these two tests. Specifically, for the full model of the endo-system gene-set, we observed an OR of 1.34 with kinship structure provided (p = 0.035) while we observed a similar OR of 1.36 assuming an independent setup (p = 0.02), which indicated that the family structure within the ADSP family study did not influence our analyses to a large extent.

To test for association with Braak stages, we first extracted only European samples with Braak staging information available for each dataset, before extracting rare deleterious SNVs for each gene within the endo-system gene-set. We leveraged the fact that it is a semi-quantitative trait and performed the association test with the SKAT function for continuous traits with the ‘optimal’ option after building null models as described for testing AD status. In the attempt to remove confounding factors and unbalanced sample distribution for Braak staging association test, we additionally included AD status in null models. Finally, we meta-analyzed variants across datasets and computed ‘Gene-level’ p-values for AD status as well as Braak staging. We combined genotyping matrices across three datasets for each gene using the R package, MetaSKAT.[90] Specifically, we first transformed our genotyping matrices into an SSD format for a single population and then analyzed all three populations at once using the function MetaSKAT_MSSD_ALL. This procedure increased the power to analyze the effects of rare variants that are shared across different studies. To correct for testing multiple genes within the endo-system gene-set, we obtained the number of genes we tested in each separate dataset and computed their corresponding Bonferroni corrected significance thresholds. Specifically, for the AD single-gene analysis, we tested 1,195, 1,228, and 683 genes in ADSP case-control, AMP-AD case-control, and ADSP family datasets, respectively, which corresponded to Bonferroni corrected significance thresholds of α = 4.18*10^−5^; 4.07*10^−5^; 7.32*10^−5^, respectively. In meta-analyses, we identified 642 genes in common and computed a Bonferroni corrected significance threshold of α = 7.79*10^−5^. For the Braak staging single-gene analysis, we retained only rare deleterious SNVs present in samples with Braak staging information available and tested for 1,035 and 1,176 genes for the ADSP and AMP-AD case-control studies, respectively. The corresponding Bonferroni corrected significance thresholds were then computed as α = 4.83*10^−5^ for the ADSP case-control dataset and 4.25*10^−5^ for the AMP-AD case-control dataset. When performing meta-analyses, we examined 967 genes in common between these two datasets, which led to a Bonferroni corrected significance threshold of α = 5.17*10^−5^.

### Functional analysis on AD

One approach to understanding how the effect of rare variants would influence the risk of AD status is to investigate how they regulate gene expression. A gene with a variation that is associated with its gene expression is called an eGene. Here, we obtained the bulk RNA-Seq data of DLPFC tissues of 636 individuals from the ROSMAP [55] study and performed an association test between the expression of a gene and rare variants in *cis* with the corresponding gene. In particular, for each gene within the endo-system gene-set, we included all variants within gene boundary and additionally all rare variants within 20kb up- and down-stream of the transcription start sites (TSS), which might potentially regulate the expression of a gene through *cis*-regulation, such as the effect of enhancer region. To overcome the problem of low power to detect the effect of single rare variants, we aggregated the effects of all rare variants within as well as near the TSS of each gene. We analyzed this aggregated effect on gene expression using the SKAT function to compute ‘Gene level’ p-values, while taking into account confounding covariates, including age, sex, sequencing locations, *APOE* ε2 and ε4 alleles, and top 10 PCs. To correct for testing multiple genes, we calculated false discovery rate for all tested genes and used FDR of 0.05 as the q-value threshold, following the suggestions of previous studies.[91,92] Follow-up validation was performed using genes previous identified from the burden and functional analyses, by directly comparing their expression levels between AD cases and controls using student t-test and computing the Pearson correlation between their expression levels and Braak stages. The multiple-testing issue was then addressed using the Bonferroni correction method.

The resolution of bulk RNA-Seq data may limit our capability of observing cell-type specific effects on AD.[55,61,93,94] To elucidate the underlying complexity of variation across cell types, we further obtained single-cell RNA-Seq (scRNA-Seq) of 48 samples (24 AD patients and 24 cognitively normal controls) from the ROSMAP study and investigated the pattern of expression for each of the six major cell types defined on a priori cell-type-specific gene-sets: excitatory neuron (Ex), inhibitory neuron (In), astrocyte (Ast), oligodendrocyte (Oli), oligodendrocyte-precursor-cell (Opc), and microglia (Mic)[61]. The six major cell types were further divided into sub-clustered cells based on the heterogeneity of gene expression within each cell type: 13 Exs, 12 Ins, 4 Asts, 5 Olis, 3 Opcs, and 4 Mics [61]. The whole dataset in 10X format was first processed using the R package, Seurat.[95] We followed the preprocessing steps as proposed by the Seurat developer by first filtering out cells with reads quantified for less than 200 or more than 2,500 genes, followed by filtering out cells with the percentage of mitochondrial gene counts over 5 percent. We then employed a global-scaling normalization method provided by the LogNormalize function, which normalized the feature expression measurements for each cell by the total expression, followed by a log-transformation. The major and sub-cell types were identified a priori for this scRNA-Seq data. Therefore, we extracted all significant genes identified in the previous single-gene and functional analyses for each specific cell type and conducted differential gene expression analysis using the student t-test method between cases and controls for each major cell type, as well as for each subcellular population within each major cell type.

## Result

### The burden of rare deleterious SNVs in endo-system gene-set for ADSP case-control study

**Fig 1:**
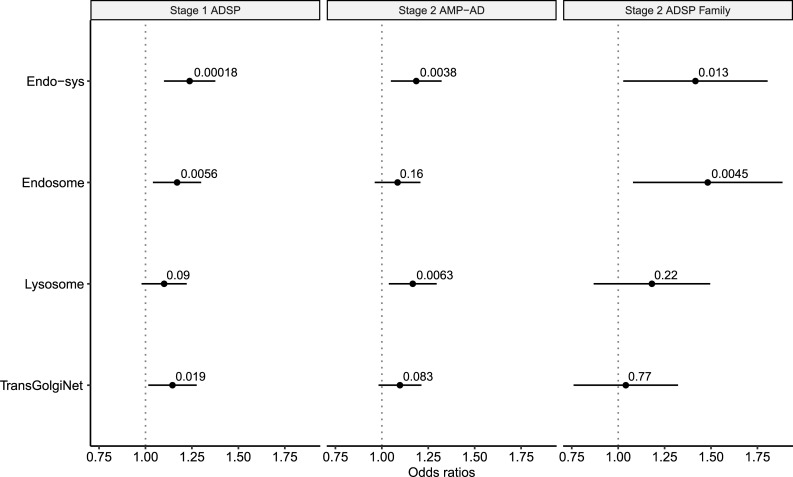
Rare deleterious variants are enriched in AD patients across the endocytic and corresponding compartmental gene-sets in stages 1 and 2. We compared the burden of rare deleterious variants between AD patients and controls across the endo-system (endo-sys) gene-set and three compartmental sub-gene-sets (endosome, lysosome, and trans-Golgi network) in stage 1 ADSP case-control dataset (leftmost), which were then tested for replication in stage 2 AMP-AD case-control (middle) and ADSP family (rightmost) datasets. Enrichment (ORs) and p-value were computed using a linear regression model controlling for covariates, including the total count of rare variants (see Methods). P-values of enrichment in each gene-set are indicated above horizontal bars which represent 95% confidence intervals.

To investigate whether rare deleterious SNVs in the endocytic pathway were associated with AD, we leveraged a gene-set method of burden analysis that collapsed individual effects of multiple variants into one ‘gene-set level’ effect, hence increasing the power of detecting rare variants’ effect. We defined rare SNVs using both an external source of allele frequency and allele frequency observed in 1,291 European samples (664 AD cases and 627 controls) from the ADSP case-control study (see Methods). We focused on deleterious SNVs as defined in Methods, in which most were protein-altering variants. We identified rare deleterious SNVs in 1,133 of the 1,435 genes in our gene-set (see Methods). For each individual, we computed the burden of these rare deleterious SNVs. We then compared the genetic burden between AD cases and cognitively normal controls, while taking into account confounding covariates that can potentially influence the amount of burden. Such covariates include ancestral principal components, age, sex, the sequencing location, the number of *APOE* e2 and e4 alleles, and the total number of rare SNVs of each individual. The last procedure is necessary to account for individual differences in the total amount of variation; an individual is likely to carry more rare deleterious SNVs if she/he carries more rare SNVs overall. To note, we found that the total number of rare SNVs on the genome-wide scale has no statistically significant difference between cases and controls (p = 0.67, student t-test). As described in Methods, we applied three logistic regression models to find associations between AD status and the burden scores while the three models were built on top of each other and tested for two MAF thresholds (1% and 0.1%). Looking at our best model in terms of the strongest association, we observed that the risk of AD, as indicated by the odds ratio (OR), increased by 1.24 for every one unit increase in residual burden score (p = 0.00018 using GLM), which was a significant association after stringent multiple testing correction (α = 0.00625) for all gene-sets (including sub-gene-sets we analyzed in next steps) (Fig 1).

Additionally, we identified three major cellular compartments participating in the endocytic pathway and their corresponding genes, which constituted subsets of the endo-system gene-set. The first two compartmental gene-sets were endosome (n = 811) and lysosome (n = 620) gene-sets, which served as the major sorting station in the endocytic pathway and the final destination of proteolytic destinations [96], respectively. The third important compartment was the trans-Golgi network gene-set (n = 208) which represented a pathway sorting station for retrograde trafficking. In summary, we identified 689 endosomal genes, 544 lysosomal genes, and 181 trans-Golgi network genes, respectively. We found the burden scores of rare deleterious SNVs were higher in cases than in controls for all three sub-gene-set. In our best model, the OR, representing the risk of AD, increased by 1.18 per unit for the endosome gene-set (p = 0.0056 using GLM), 1.08 per unit for the lysosome gene-set (p = 0.09 using GLM; Fig 1), and 1.14 per unit for the trans-Golgi network gene-set (p = 0.019 using GLM). After the multiple-testing correction, we observed the endosome gene-set showed a gene-set-wide significant association with AD while the trans-Golgi network displayed a nominally significant association signal. In addition to exploring sub-gene-sets, we also checked the specificity of the association in the endocytic pathway by obtaining gene-sets unrelated to AD. Specifically, we explored two non-disease complex traits, BMI and height, and obtained related genes (212 and 78, respectively) from GeneRIF, a publically available database for functional annotations.[97] Indeed, we did not observe an enriched rare-variant burden in AD cases compared to controls in these gene-sets and the directions of effects were different across datasets, suggesting the observed rare-variant effect was specific to the endocytic pathway (S13 Table).

### Stage 2 replication of the burden analysis in two independent WGS datasets

The gene-set burden analysis in the ADSP case-control study demonstrated statistically significant enrichment of rare deleterious SNVs in cases in the endocytic pathway, indicating an increase of risk conferring AD. We further examined the endo-system gene-set in 1,611 European samples (642 AD cases and 969 controls) from the AMP-AD study. We obtained 1,198 endo-system-related genes and observed an elevated risk of AD in terms of OR of 1.19 (p = 0.0038 using GLM; Fig 1), replicating the observation of a significantly higher burden of rare deleterious SNVs in the stage 1 analysis, using a multiple testing threshold of α = 0.00625.

We performed additional gene-set burden analysis on the sub-gene-sets of the functional compartments in the AMP-AD study. We identified 735 endosomal genes, 576 lysosomal genes, and 187 trans-Golgi network genes, respectively. We again observed an increase in AD risk among cases for all three sub-gene-sets. A nearly significant signal was observed in the lysosome gene-set with an OR of 1.17 (p = 0.0063 using GLM; Fig 1). For the other two gene-sets, we observed an OR of 1.08 (p = 0.16 using GLM; endosome gene-set) and 1.10 (p = 0.083 using GLM; trans-Golgi network). None of these gene-sets showed gene-set-wide significant association after multiple testing correction at α = 0.00625, although the lysosome gene-set nearly reached the gene-set-wide significance threshold.

As described above, the AMP-AD study consisted of three sub-cohorts and the largest one, ROSMAP, contained around 71.5% of the total sample size. To avoid potential batch effect diluting the association signal, we re-performed the analysis on only the ROSMAP data. In fact, we observed slightly more significant results in nearly all gene-sets, where the endo-system and the lysosome gene-sets both reached gene-set-wide significance threshold. (S2 Table) Overall, the associations were similar between the AMP-AD and ROSMAP data, indicating a relatively low level of batch effect among the three sub-cohorts.

Given the observed risk in stage 1 ADSP case-control study and the stage 2 AMP-AD replication, we further examined the genetic burden in the ADSP Family study. We filtered and annotated rare deleterious SNVs based on the same workflow using 353 European samples (144 AD cases and 209 controls) of the ADSP family study. Due to the smaller sample size compared to the previous two case-control studies, we obtained 683 endo-system-related genes. To examine the AD risk, we performed GLMM using the burden of each individual. Due to family structure, we utilized the generalized linear mixed model to account for the relatedness between samples. We observed an OR of 1.42 (p = 0.013 using GLMM), conferring an elevated AD risk among cases compared to controls. (Fig 1) This observation was not gene-set-wide significant using the Bonferroni correction threshold at α = 0.00625. However, it displayed a nominally significant association with the same direction of effect as in the ADSP and AMP-AD case-control studies.

Nonetheless, we looked into the sub-gene-sets of the three functional compartments in the ADSP family dataset. We identified 402 endosomal genes, 342 lysosomal genes, and 106 trans-Golgi network genes, respectively. We observed a significant elevation of AD risk among cases for endosome gene-set with an OR of 1.48 (p = 0.0045 using GLM). Similar increases were also observed in the lysosome and trans-Golgi network gene-sets, with OR of 1.18 (p = 0.22 using GLMM) and 1.04 (p = 0.77 using GLMM), respectively (Fig 1). Only the endosome gene-set remained gene-set-wide significant after multiple-testing correction, which was in concordance with our observation in the stage 1 ADSP case-control study.

### A meta-analysis of stage 1 and 2 burden analysis

The stage 1 burden analysis using the ADSP case-control study demonstrated a significant increase in AD risk in the endo-system gene-set, which was replicated in one independent dataset, the AMP-AD case-control dataset, and displayed a nominal significance in the ADSP family study. We meta-analyzed the results using two different methods (see Methods) and computed a ‘Gene-set level’ p-value of 2.17*10^−7^ (by METAL; Fisher’s method produced similar results; Table 2) for the endo-system gene-set, which was improved compared to stages 1 and 2. The same was also observed for sub-gene-sets where we computed a meta-analysis p-value of 9.78*10^−5^ for the endosome gene-set, 9.83*10^−4^ for the lysosome gene-set, and 1.19*10^−2^ for the trans-Golgi network gene-set. Except for the trans-Golgi network gene-set that has the smallest number of genes, all other gene-sets remained gene-set-wide significant after multiple-testing correction (α = 0.00625), which strongly demonstrated a shared effect of rare deleterious variants within the endocytic pathway across multiple independent studies. To note, although we meta-analyzed the results from the best models, as proposed by Zhang et al. to improve the power of detection, the same pattern of rare-variant association could be observed using the same models for each gene-set across the three datasets. (S1 Table) For all models in the endo-system, endosome, and lysosome gene-sets except M2 of lysosome, we observed gene-set-wide significant p-values, regardless of the meta-analysis methods used, demonstrating a high consistency with the observations made using the best models.

**Table 2:** Meta-analysis of stages 1 and 2 gene-set burden analyses using AD, NFT, and AAO.

Phenotype	AD		NFT		AAO	
	P	P*	P	P*	P	P*
Endo-system	2.17E‐07	2.66E‐07	1.16E‐02	9.89E‐03	2.47E‐06	4.93E‐07
Endosome	9.68E‐05	6.05E‐05	1.30E-01	9.34E-02	3.33E‐05	2.04E‐05
Lysosome	9.83E‐04	1.15E‐03	6.56E‐03	6.11E‐03	1.10E‐02	3.11E‐04
TransGolgiNet	1.20E‐02	7.46E‐03	5.71E-01	3.53E-01	2.10E‐02	4.96E‐03

Abbreviations: AD: Alzheimer’s disease; NFT: neurofibrillary tangle; AAO: age-at-onset

NFTs were analyzed using Braak stages. Gene-set-wide significant results were highlighted in bold. Displayed results of gene-set burden analyses were each meta-analyzed using METAL (P) and Fisher’s method (P*) (see Methods). Directions of effects were consistent across all tests.

A similar pattern of meta-analysis results was also observed in the additional validation tests from two aspects. Firstly, we wanted to check our results using different annotation tools. Given the set of deleterious variants used in previous phase 1 analyses, we additionally filtered by CADD scores (see Methods) and re-ran the gene-set AD association analyses with the resulting set of pathogenic/deleterious variants. In the meta-analysis, we observed that the endocytic, endosome, and lysosome gene-sets reached gene-set-wide significance threshold (see S5 Table), consistent with the rare-variant effect we observed in the endocytic pathway using the original set of rare deleterious variants.

The second aimed to validate our gene-set burden analysis using MAGMA with two different aggregation methods (see Methods). In the meta-analysis, both the SNP-wise and burden methods provided gene-set-wide significant self-contained p-values for nearly all gene-sets (S3 and S4 Tables; for endo-system, SNP-wise: 9.28*10^−7^; burden: 5.16*10^−8^), similar to the results shown above in Table 2. Compared to the MAGMA burden method, the SNP-wise method was not designed for rare-variant-only analysis and indeed showed weaker association signals. Especially for the competitive p-values, we observed gene-set-wide significant results for nearly all gene-sets using the MAGMA burden method, but not the SNP-wise method (for endo-system, SNP-wise: 2.41*10^−2^; burden: 1.90*10^−3^). We also attempted to compute a weighted burden score using pLI scores by PLINK and observed gene-set-wide significant associations in the endo-system gene-set in the meta-analysis. (S10 Fig, S12 Table) Compared to our main method above, the MAGMA methods and the weighted method displayed some fluctuations in individual datasets and models but consistent results in meta-analysis, indicating a robust rare-variant effect in the endocytic pathway under different statistical methods. Besides, as *APOE* was a major risk determinant in AD, in this validation, we also checked whether our observed rare-variant enrichment was mainly contributed from this gene, rather than the whole endo-system gene-set, by re-run the analysis with *APOE* excluded. Indeed, we observed nearly the same p-values in the meta-analysis, indicating a rare-variant enrichment in AD cases even without *APOE*.

### The burden of rare deleterious SNVs on NFTs

NFT, measured in Braak staging, was one of the most important histopathological indicators of AD [48–50]. It is designed as an ordinal scale from 0 to VI of NFT pathology where AD patients with high Braak stages (V or VI) are diagnosed with high confidence.[98] Therefore, Braak stages may serve as a finer spectrum or proxy of AD severity and provide higher power in assessing the effect of rare variants in AD progression. Based on our previous AD analysis, we hypothesized that the burden of rare deleterious variants in the endocytic pathway would be higher in patients with later Braak stages. To test our hypothesis, we applied an ordinal logistic regression (OLR) method to Braak stages (see Methods). This method has been previously shown to be effective in studies of Braak staging as well as of other ordered phenotypes, such as oral cancers.[99,100] We obtained 626 individuals (475 AD cases and 151 cognitively normal controls) from the stage 1 ADSP case-control dataset and 1,399 individuals (533 AD patients and 866 controls) from AMP-AD case-control dataset with Braak staging information, which were used to fitted OLR models. In stage 1, We observed an OR of 1.16 (p = 0.039 using OLR; S1 Fig) in the endocytic pathway, implicating a nominally significant association of rare-variant enrichment to later Braak stages. However, this result did not replicate in stage 2 with sufficient significance (OR = 1.08, p = 0.13 using OLR; S1 Fig). Comparing the stages 1 and 2 samples, we observed a distinct distribution of Braak stages. In particular, the stage 2 samples were concentrated in Braak stage III (23.1%), IV (28.1%), and V (23.3%), whereas most stage 1 samples were clustered in stage V (26.4%) and VI (34.8). (S3 Fig) We did not test for replication in the ADSP Family study due to limited samples with Braak staging information (n = 38 individuals where only one sample had AD).

Our analyses of two independent datasets suggested a trend of increased risk of bearing later Braak stages with elevated rare-variant burden in the endocytic pathway. To improve power, we meta-analyzed the results from the ADSP and AMP-AD case-control studies, producing a ‘Gene-set level’ p-value between 0.0099 and 0.012, which did not pass our multiple-testing correction threshold of α = 0.00625. (Table 2) Further Braak staging burden analysis using compartmental sub-gene-sets, however, revealed a gene-set-wide significant signal in the meta-analysis for lysosome gene-set (p = 0.0066, Fisher’s method). A full list of results for NFT burden analysis can be found in S6 Table.

### Hazard analysis on population risk of AD age of onset and death

**Fig 2:**
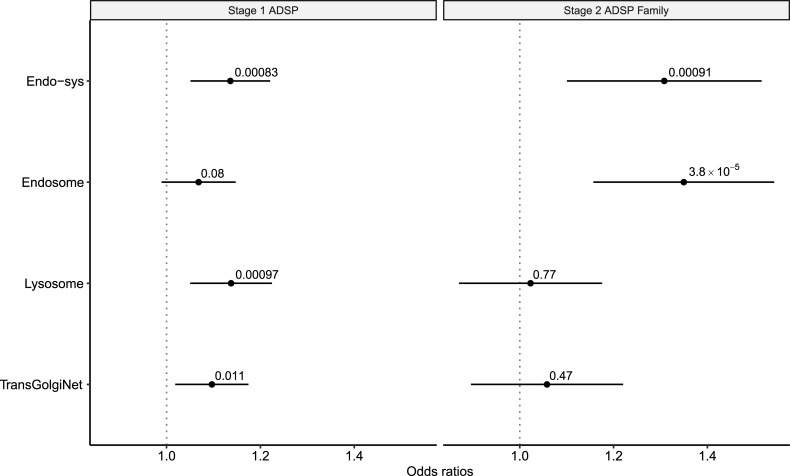
The enrichment of rare deleterious variants is associated with AD AAO across the endocytic and corresponding compartmental gene-sets in stages 1 and 2. We computed a hazard ratio of obtaining AD in earlier ages using the burden of rare deleterious variants across the endo-system gene-set and three compartmental sub-gene-sets (endosome, lysosome, and trans-Golgi network) in stage 1 ADSP case-control dataset (left), which were then tested for replication in stage 2 ADSP family datasets (right). Enrichment (ORs) and p-value were computed using CPHR. P-values of enrichment in each gene-set are indicated above horizontal bars which represent 95% confidence intervals.

Previous gene-set burden analyses have demonstrated a significant correlation between the burden of rare deleterious variants within the endocytic gene-set and AD risk. One important aspect of AD development is its age-specific phenotypes, such as AAO. Previous studies on AD have shown a large genetic component in the heritability of AAO [101,102], with multiple risk loci associated with it. [103–107] It is thus of interest to also examine the genetic risk identified within the endocytic gene-set in this context. One approach is to evaluate whether AD patients with earlier AAO are associated with greater rare-variant burden within the endocytic gene-set. Previous studies have proposed a genetic epidemiological framework, where age-specific phenotypes were analyzed using a Cox Proportional Hazard Regression (CPHR) that considered a time-to-event probability, as opposed to the simple event probability estimated in logistic regression.[86,108] Therefore, we leveraged our previously computed burden score for each individual in the ADSP case-control study and constructed a cox proportional hazard (CPHR) model to estimate the instantaneous risk of developing AD, in consideration of genotype and AAO. A positive estimate of hazard in this model would indicate a higher risk of developing AD in early ages. We built three models as in the burden analysis and observed in our best model that an AAO-specific genetic risk increased by 1.14 per unit increase in the residual burden score (p = 0.00083 using CPHR; Fig 2), which reached gene-set-wide significance after multiple testing correction (α = 0.00625). We further examined the AAO-specific genetic risk within the functional sub-gene-sets. In our best model, we observed a gene-set-wide significant hazard ratio of 1.14 (p = 0.00097 using CPHR) for lysosome gene-set and a nominal significant hazard ratio of 1.10 (p = 0.011 using CPHR) for trans-Golgi network gene-set.

To test for replication, we examined the ADSP family study under the same statistical framework. Applying the CPHR models, we observed a gene-set-wide significant hazard ratio of 1.31 (p = 0.00091 using CPHR; Fig 2) in the endo-system gene-set. Carefully examining the sub-gene-sets also revealed gene-set-wide significant AAO-specific risk within the endosome gene-set (HR = 1.35, p = 3.83*10^−5^ using CPHR). We did not observe significant associations using the other two compartmental gene-sets (S7 Table).

To increase power, we performed meta-analyses to identify rare-variant effects shared across multiple studies. We combined the best results from ADSP case-control and family studies and observed a gene-set-wide significant p-value of 2.47*10^−6^ (by METAL; Fisher’s method produced similar results; Table 2) for the endo-system gene-set, which was greatly improved compared to results in either stage. Similarly, the endosome gene-set also demonstrated an improved gene-set-wide significant p-value of 3.33*10^−5^. However, the lysosome and the trans-Golgi network gene-sets showed only nominally significant p-values in our meta-analysis, potentially due to the absence of signal in the ADSP family study. These findings strongly demonstrated that this AAO-specific rare-variant effect in the endocytic pathway was shared in European samples across different studies.

Another age-specific phenotype is the age of death (AOD), which has been shown to be affected by genetic groups implicated in AD AAO as well as in other dementia.[109,110] We thus followed the same analysis framework using the CPHR model and assessed whether AD-affected patients with earlier AOD were associated with a higher rare-variant burden in the endocytic pathway. We looked at European samples in the AMP-AD case-control study, where the AOD information was available. We observed a hazard ratio of 1.10 (p = 0.024 using CPHR; S2 Fig), indicating an increase of risk of death in AD patients as well as a worse prognosis along with an elevation in genetic burden. Further analysis using the lysosome sub-gene-set displayed a hazard ratio of 1.09 (p = 0.036 using CPHR). Both endo-system and lysosome gene-sets demonstrated nominally significant associations with AOD but did not reach gene-set-wide significance after multiple-testing correction. Analysis using other sub-gene-sets did not provide significant hazard ratios.

### Single-gene analysis on AD risk using AD and NFT status

From the previous analysis, the endo-system gene-set conferred a large rare-variant effect on AD and related phenotypes. Thus, we decided to examine the effect of rare variants in single endocytic genes, attempting to identify those associated with AD with large effect sizes. To increase power, we aggregated previously defined rare deleterious SNVs in each gene and tested for association with AD. We did not observe a single gene passing the Bonferroni corrected significance threshold in all three datasets, as well as in meta-analysis (See Methods; α = 4.18*10^−5^; 4.07*10^−5^; 7.32*10^−5^; 7.79*19^−5^, for ADSP case-control, AMP-AD case-control, ADSP family studies, and meta-analysis respectively; S15 Table).

As mentioned previously, NFT status may provide more detailed information of the pathological progression of AD and thus a greater power to detect signals of rare-variant effect. We, therefore, performed single-gene analysis using NFT status, as a proxy for AD status. For all datasets, we retained only rare deleterious SNVs that were present in samples with Braak staging information. We controlled for the same set of covariates as in previous analyses, except that we also included the AD phenotype (AD affected/unaffected) for each individual as one additional covariate (see Methods). The latter is necessary because the Braak staging and the AD phenotype are correlated, and the numbers of individuals with and without AD were vastly disproportionate among the samples with Braak staging information. For the ADSP case-control study, we observed six genes that reached Bonferroni corrected significance threshold (α = 4.83*10^−5^). None of the genes passed the Bonferroni corrected significance threshold (α = 4.25*10^−5^) in the AMP-AD study. Results of the top ten most significant genes can be found in S8 Table. We conducted meta-analyses for these two independent studies using MetaSKAT as before. In the combined results, we observed one gene, *ANKRD13D*, reached Bonferroni corrected significance threshold (p = 3.56e-05; α = 5.17*10^−5^). This gene has been previously implicated in AD through RNA expression analysis [111] and protein interactome mapping [112].

### The identification of functional effects of rare variants within the endocytic pathway

**Fig 3:**
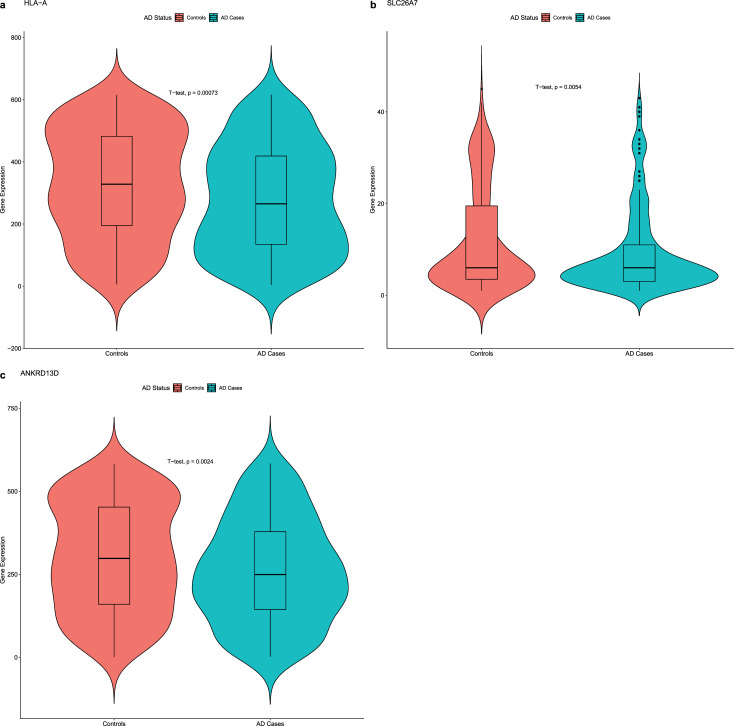
Comparison of the gene expression of *HLA-A*, *SLC26A7*, and *ANKRD13D* between AD cases and controls from the ROSMAP study. Violin plots were used to represent the distribution of gene expression within each AD status, where a symmetric deviation from the middle line on both sides indicated a higher abundance of samples at the corresponding gene expression level. Comparisons between AD cases and controls were assessed using boxplots. P-values were computed using the student t-test. All three genes, *HLA-A*, *SLC26A7*, and *ANKRD13D*, are down-regulated in AD cases compared to controls.

The hypothesis that the endo-system gene-set contains rare variants that are influential to AD development is endorsed by the previous gene-set burden analyses and single-gene analyses. One approach to investigating how the effect of rare variants takes place is to analyze how these rare variants are associated with gene expression. Such gene containing variations affecting its expression is often called an eGene.[91] To identify eGenes, we obtained bulk RNA-Seq data of DLPFC brain tissues of 636 individuals from the ROSMAP study as part of the AMP-AD study and tested for association of all variants in *cis* with a gene with its gene expression. Specifically, we grouped all variants within one gene, as well as those near the corresponding TSS, and assessed whether the aggregated rare-variant effect in an endocytic gene is associated with its expression level using SKAT (see Methods). Intersecting the bulk RNA-Seq and WGS data revealed 547 individuals with 224 AD patients and 323 controls. By taking an FDR of 5%, we discovered two genes, *HLA-A* and *SLC26A7*, whose rare variants were significantly associated with expressional changes. To note, previous studies have demonstrated that proteins from the same families of these two genes are associated with AD status. Specifically, two proteins from the HLA families and one from the SLC families have been implicated in AD through meta-analyses of large GWAS and brain DNA-methylation association analysis.[9,113] We first examined their single-gene analysis results and observed that none of them was significant using the AMP-AD dataset (p = 4.74e-01; 2.14e-01, for *HLA-A* and *SLC26A7*, respectively). To validate our results and determine the direction of effects, we compared the expression of these two genes between cases and controls. Indeed, their expression levels were both significantly decreased in cases compared to controls (p = 0.00073 *HLA-A*; Fig 3A; p = 0.0054 *SLC26A7*; Fig 3B; student t-test; α = 0.017;). We further examined the distribution of their expression levels across multiple Braak stages. Similarly, both expressions were strongly negatively correlated with greater Braak stages (r = -0.129, p = 0.0024 *HLA-*A; r = -0.127, p = 0.0029 *SLC26A7*; Pearson correlation; α = 0.017).

We also investigated *ANKRD13D*, which we previously identified to be associated with Braak stages, in the context of gene expression. Although not an eGene, *ANKRD13D* exhibited a significant expressional decrease in cases compared to controls (p = 0.0026; student t-test; Fig 3C). The analysis on Braak staging also revealed a strong negative correlation (r = -0.122, p = 0.0042; Pearson correlation).

### scRNA expression analysis

Recent advancement in analyzing gene expression in single-cell resolution has provided opportunities to uncover complex alterations across cell types and identify cell-type specific effects on AD.[55,61,93,94] For example, previous studies have pointed the imbalance of excitatory and inhibitory neurons could lead to overexcitability and early dysregulation in the development of AD [114]. Many other studies also demonstrated abnormalities in innate immune cells, primarily microglia, in the pathogenesis of AD.[115] Therefore, to investigate the potential cell-type specific effects of rare variants within the endocytic pathway, we obtained the single-cell RNA-seq data of 48 samples (24 AD patients and 24 cognitively normal controls) from the ROSMAP study. We focused on three genes we identified through the previous analysis, which demonstrated significant associations to AD progression. The scRNA-Seq data were labeled with six major cell types using a priori marker genes (Ex, In, Ast, Oli, Mic, and Opc), and sub-clustering within each cell type revealed cellular subpopulation (see Methods). We examined the expression of the three target genes in all major cell types and observed that *ANKRD13D* was up-regulated in Ex (p = 1.92*10–18; student t-test), Ast (p = 0.011; student t-test), and In (p = 0.028; student t-test) (S9 Table). However, it exhibited a down-regulation in Oli (p = 0.0018; student t-test). *SLC26A7* was observed to be up-regulated in Ex (p = 0.0049; student t-test), while *HLA-A* displayed a pattern of down-regulation in both In and Mic (p = 9.72*10-6and p = 0.0031 respectively; student t-test). Four AD pathology-associated cellular subpopulations (Ex4, In0, Ast1, and Oli0) have been previous demonstrated for this scRNA-Seq data [61,91]. Our differential expression analysis within these four subpopulations showed a pattern of up-regulation of *ANKRD13D* in Ex4 and In0 (p = 5.76*10–8 and p = 0.036, respectively; student t-test; S10 Table). The other two genes, however, were not significantly differentially expressed in these four cell subpopulations.

## Discussion

Using large publicly available WGS datasets, our study described here enabled us to assess the contribution of rare variants to AD. In our stage 1 discovery phase, we observed a significantly elevated burden of rare deleterious SNVs in affected individuals compared to cognitively normal older controls within the endocytic pathway. We chose this pathway because it represented one of the earliest morphological changes in AD development, and multiple AD risk factors, predominantly through common SNPs, have been implicated specifically in this pathway with genome-wide significance, including *BIN1*, *CD2AP*, *PICALM*, *RIN3*, and *SORL1*.[9,12,18,19,21,22,116] Our results demonstrated additional correlation between rare variants in the endocytic pathway and AD. Successful replication in the AMP-AD case-control study and improved meta-analysis association further strengthened this contribution of rare deleterious variants to AD risk. Our analysis using the ADSP family dataset showed a similar enrichment of rare deleterious SNVs in AD patients, although not reaching gene-set-wide significance. One possible explanation was that the sample size of this family study was relatively small (one third to one fifth) compared to the other two case-control studies. We additionally identified gene-set-wide-significant signals within the endosome and lysosome gene-sets using meta-analysis, implicating potential compartment-specific roles in AD pathology. One possibility that we did not observe significant results in separate stages for all three sub-gene-sets was because they contained a smaller number of genes compared to the endo-system gene-set and, therefore, smaller aggregated effects of rare variants, which required meta-analysis to combine signals in individual samples. As the smallest gene-set (one-third to one-fourth of the other two), the trans-Golgi network remained nominally significant even after meta-analysis.

In assessing the AD pathological progression, we examined the association of rare-variant effect to NFT pathology using Braak staging. We observed the gene-set-wide significant association within the lysosome gene-set, where individuals with higher Braak stages were enriched with rare deleterious SNVs. No significant association was found in other gene-sets, besides a nominally significant association in the whole endocytic pathway. Compared to the previous analysis using AD status, our analysis using Braak stages was largely limited in sample size. For example, only 626 out of 1,291 European samples in the ADSP case-control dataset had Braak staging information available. For the ADSP family study, only 38 out of 353 samples had Braak information available, which made analyzing Braak stages in this dataset infeasible. Additionally, this was further complicated by the disproportionate distribution of samples across different Braak stages. The ADSP case-control dataset contained 218 samples in stage VI while only 15 samples in stage 0. Such highly skewed distribution reduced our power to detect a significant association between rare variants’ effects and Braak stages. The AMP-AD dataset was similarly skewed but also distributed largely differently from the stage 1 dataset. This distinction in distribution may explain why we observed different signals in our stage 1 and 2 analyses.

Based on the idea that rare variants within the endocytic pathway were associated with AD progression, we further tested age-specific phenotypes and leveraged a CHPR model previously proposed to be effective in assessing the effect of variants on age-to-event risk.[86] For AAO, we observed a gene-set-wide significant hazard ratio in the stage 1 analysis, indicating an association of rare-variant burden in the endocytic pathway to earlier AAO of AD, which was replicated in stage 2. A similar observation was found in the compartmental gene-sets, where endosome gene-set demonstrated a gene-set-wide significant signal in meta-analysis. Nonetheless, we did not replicate our stage 1 findings of the lysosome gene-set in stage 2, potentially due to the small sample size of the family dataset and the small size of the gene-set. For AOD, we examined the AMP-AD dataset and only observed nominally significant signals in the endocytic pathway and the lysosomal compartment. Previous analyses on AAO have demonstrated a substantial correlation of AAO between parents and their children, with multiple risk loci, such as *APOE*, *GRN*, *MPT*, and *C9orf72*.[101,109] Genetic studies using AOD from LOAD datasets have revealed additional associations of SNVs in these genes with human aging.[110] Consistent in the observation of significant genetic components, our results discovered an additional contribution of rare variants within the endocytic pathway to age-related phenotypes.

Our discovery of the increased burden of rare-variant effect in AD patients led us to explore the effect of individual genes within the endocytic pathway and attempt to identify specific ones with large effect sizes which might serve as potential clinical and therapeutic targets. We performed single-gene analysis using both AD status and Braak staging as the target phenotypes. When looking at the AD status, we did not observe a gene with a large enough effect to be detected in our analysis. Using Braak staging information, we were able to identify one gene, *ANKRD13D*, that showed robust signal across multiple studies after multiple-testing correction. This may be due to the fact that Braak stages provided a finer indication of AD progression. *ANKRD13D* encodes a member of the Ankyrin repeat domain 13 family, characterized by three ankyrin repeats at the N-terminal facilitating protein-protein interaction.[117] It has been experimentally shown to localize to endosomes and is known to regulate the rapid ubiquitin-dependent internalization and sorting of membrane-bound proteins within the endocytic pathway.[118] One of its main targets is the endocytosis of the epidermal growth factor receptor (EGFR) through the functional ubiquitin-interacting motif (UIM) of the ANKRD13 family proteins, which is then degraded in lysosomes.[118,119] EGFR is a transmembrane protein serving as a receptor epidermal growth factor (EGF) protein ligands. Multiple previous studies have reported abnormal plasma levels of EGF in AD patients, [120–122] and two recent studies on EGF have demonstrated its protective effects on AD by preventing amyloid-beta (Aβ)-induced angiogenesis deficit to brain endothelial cells in vitro and in vivo.[123,124] Recent studies have also described that the EGFR internalization after EGF binding was strongly inhibited when ANKRD13 proteins were over-expressed.[118] This mechanism implicates a potential regulatory effect of the *ANKRD13* family on AD pathology through the regulation of internalization of EGFR. Indeed, the link between *ANKRD13D* and AD is further bolstered by a recent RNA profiling where they identified an altered gene expression of *ANKRD13D* between the blood and brain tissue of AD patients.[111] In our analysis, we identified seven rare deleterious SNVs within *ANKRD13D*, where six were predicted to be missense damaging variants and one was predicted to be either missense damaging or splice region variant. These mutations could potentially alter its ubiquitin-binding ability, either through directly changing the sequence or indirectly through changing the 3D protein folding structure, and affect the normal protective function of EGF in AD development. Further functional studies of *ANKRD13D*, and in particular these seven variants, will be needed to specifically define its role in AD pathogenesis and evaluate the therapeutic and clinical importance of the EGFR pathway.

To investigate the functional effects of rare variants, we looked at the expression of genes in the endocytic pathway at both bulk tissue and single-cell resolutions. Leveraging bulk RNA-Seq data, we identified two significant eGenes, *HLA-A* and *SLC26A7*, in the ROSMAP study. Careful examination of these two eGenes in the context of AD status revealed a pattern of down-regulation in AD patients compared to cognitively normal controls. A similar negative correlation was found using Braak stages. *HLA-A* encodes a member of the human leukocyte antigen A (HLA) class I, also called the major histocompatibility complex (MHC) class I. It has been shown to participate in the important “cross-presentation” mechanism of T cell-mediated immune response, specifically efficient in dendritic cells.[125] This mechanism is part of the endocytic pathway that involves the internalization of HLA class I proteins from the cell surface through early endosomes and the loading of antigen peptides in lysosomes.[126] Previous studies have described an important role of HLA class I in maintaining the integrity of aging brains and have demonstrated significant dendritic atrophy with deficient HLA class I.[127] Moreover, recent GWA studies have identified specific alleles in *HLA-A* associated with AD in the Italian and Chinese population [128,129], as well as risk loci in other members of the HLA family.[9] The other identified eGene, *SLC26A7*, encodes a member of the solute carrier (SLC) family that localizes to subapical lysosomal membrane as well as endosomes, primarily serving as an exchanger and transporter of a broad spectrum of substrates in the endocytic pathway.[130,131] Disruption in the expression of SLC26 proteins has been shown to cause severe acid-base balance dysregulation, leading to disruption of anion homeostasis.[132] Multiple SLCs have been associated with AD, such as *SLC2A2*, which was linked to astrocyte activation leading to its elevation in AD patients, [130] and *SLC1A3*, whose expression has been associated with Aβ deposition [133]. Recent GWA studies have also identified risk loci in members of *SLCs*, such as *SLC24A4*.[9] Specific implication of *SLC26A7* has also been shown through gene co-expression network mining where STAT1, a transcription factor of *SLC26A7*, was differentially expressed between AD patients and cognitively normal controls.[47] In our analysis, we identified nine rare deleterious SNVs in *HLA-A* in which six were predicted to be damaging missense mutations, two were predicted to be splice acceptor variants, and one was predicted to be either damaging missense mutation or splice region variant. In *SLC26A7*, we also identified nine rare deleterious SNVs, which are all damaging missense mutations. As transporters, these two genes could potentially be altered in their affinities to ligands due to changes in primary or tertiary structures. Our results here supported these previous findings and provided additional evidence from the aspect of the rare-variant effect on gene expression. Further investigation will be required to elucidate specific variants conferring these effects as well as other participating proteins in the same signal relay mechanisms of *HLA-A* and *SLC26A7*.

In a single-cell resolution, we further explored the cell-type-specific functional effects of the significant genes identified in our previous analyses. Previous single-cell transcriptomic analyses have shown a large number of cellular subpopulations with cell type-specific associations with AD.[61] Our analysis supported this finding in *ANKRD13D*, *HLA-A*, and *SLC26A7*. For example, we observed an up-regulation of *ANKRD13D* in bulk tissue, but it was found to be regulated differently in different cell types: up-regulated in Ex, Ast, and In, while down-regulated in Oli. On the other hand, in single-cell RNA-Seq data, *SLC26A7* and *HLA-A* showed a pattern of down-regulation in AD patients, consistent with our findings using the bulk RNA-Seq data though with various effect sizes in different cell types.

Several strengths and limitations of our study warrant discussion. One of the major strengths is our study design to begin the analysis with pathways implicated in AD a priori. Our usage of the endocytic pathway provided us the power to identify rare-variant effects that would otherwise be missed in traditional association analysis of single variants. This design was further combined with the large sample sizes of the three independent datasets, which provided additional power. We separated these datasets into a discovery phase and a replication phase and were able to replicate our discovery phase results in two independent datasets of the replication phase, followed by meta-analyses of samples in all three studies. This procedure ensured us to identify and validate associations while retaining large power to identify small signals. Another strength of our study is the analysis of AD-related phenotypes, such as Braak stages, and provided additional power in identifying single genes with large aggregated rare-variant effect sizes. The analysis of AAO and AOD provided further information on the progression of AD, which is especially important in clinal AD prediction and intervention. One more strength in our analysis lies in our exploitation of bulk- and sc-RNA expression data in combination with AD genotyping data. Through this method, we were able to identify eGenes with large rare-variant effect, which would require a much higher sample size and greater power to be identified as eQTLs and suggested potential AD-regulating mechanisms.

One limitation of the study is that while we used WGS datasets, we only focused on analyzing rare SNVs within genic regions. Our analysis relied on knowing the deleteriousness of each variant contributing to the gene-set burden, and variant annotation is most reliably predicted for coding and splice site variants.[90,134] Including variants in intergenic regions or indels may result in the inclusion of variants with benign effects and decrease our power of detecting AD-associated genetic burden. Another limitation of our study is that even though we utilized WGS datasets of large sample size, they were not large enough to detect single genes where rare variants significantly influenced AD. Although our analyses displayed sufficient power to detect rare-variant effects within sets of genes, we nonetheless failed to directly identify direct gene-level associations with AD. To achieve this latter goal, we may need WGS datasets of larger sample sizes. A similar limitation on sample sizes was seen in those with expression data and Braak staging information. Our bulk RNA-Seq data is only available for 547 individuals from the ROSMAP study in which we have genotyping data for 1200 individuals. The scRNA-Seq data is further limited in that we have 48 samples from the ROSMAP study. These limitations in sample size decreased our capability of detecting functional effects of rare variants within the endocytic pathway. One more limitation in this study is that we primarily focused on European samples because we had a limited sample size for non-European ancestries across all three WGS datasets. Nonetheless, it may be of interest to check whether we would observe similar rare-variant effect in the endocytic pathway in non-European samples as we observed in European samples. Another limitation rooted in the potential batch effects among the ADSP datasets used in this study, as also mentioned in Holstege et al.[135], due to the fact that the samples were sequenced and called in different locations. In this study, we have addressed the potential batch effect from three aspects. Firstly, the version of the ADSP datasets used in this study has been quality controlled, where all samples from different centers were re-processed using the same VCPA 1.0 pipeline and corrected for many technical issues present in the previous version, including contaminations, mismatches, and duplicates.[136] Secondly, we conducted additional QC steps at variant-level and sample-level. These included many steps suggested by Holstege et al., such as sex-check, selecting European samples by PCA, removing unexpected related samples using IBD, checking for samples with aberrant Ti/Tv ratio or novel SNV/indel count, and filtering out variants failing VQSR, GQ, HWE, and missing rate thresholds. Thirdly, we included sequencing location as a covariate in all models (M0, M1, and M2) to account for potential batch effects. Therefore, in this study, we recognized and have carefully approached this limitation, as much as we could, to mitigate the potential batch effects.

In summary, our study demonstrated significant rare-variant effect within the endocytic pathway in European samples. Such effect was also associated with Braak stages and age-related phenotypes, suggesting a potential target for clinical and therapeutic studies. Further investigation within this pathway revealed one gene significantly associated with Braak stages and two eGenes with a pattern of differential expression between AD patients and cognitively normal controls. More functional studies will be necessary to gain a better understanding of their molecular mechanisms of how they participate in the processing and modification of AD-related proteins. In vitro and in vivo experiments on these genes will also provide further insights into the connections of genetic variants to their gene expression and elucidate protein signaling models that affect the pathogenic progression of AD.

## Supporting information

S1 Fig Rare deleterious variants are enriched in patients with severe NFTs across the endocytic and corresponding compartmental gene-sets in stages 1 and 2. We compared the burden of rare deleterious variants between patients with different severity of NFT across the endo-system gene-set and three compartmental sub-gene-sets (endosome, lysosome, and trans-Golgi network) in stage 1 ADSP case-control dataset (left), which were then tested for replication in stage 2 AMP-AD case-control dataset (right). Enrichment (ORs) and p-value were computed using OLR controlling for covariates, including the total count of rare variants (see Methods). P-values of enrichment in each gene-set are indicated above horizontal bars which represent 95% confidence intervals.
(EPS)

S2 Fig The enrichment of rare deleterious variants is associated with AD AOD across the endocytic and corresponding compartmental gene-sets. We computed a hazard ratio of earlier AOD with AD using the burden of rare deleterious variants across the endo-system gene-set and three compartmental sub-gene-sets (endosome, lysosome, and trans-Golgi network) in the AMP-AD study. Enrichment (ORs) and p-value were computed using CPHR controlling for covariates, including the total count of rare variants (see Methods). P-values of enrichment in each gene-set are indicated above horizontal bars which represent 95% confidence intervals.
(EPS)

S3 Fig Distribution of Braak stages in individuals from Stage 1 ADSP and Stage 2 AMP-AD datasets. (EPS)

S4 Fig PCA plots (PC1 vs. PC2) of the ADSP case-control dataset showing the distribution of ancestry backgrounds. (EPS)

S5 Fig PCA plots (PC1 vs. PC2) of the AMP-AD case-control dataset showing the distribution of ancestry backgrounds. (EPS)

S6 Fig PCA plots (PC1 vs. PC2) of the ADSP Family dataset showing the distribution of ancestry backgrounds. (EPS)

S7 Fig Overlapping genes between gene-sets (the endocytic, the immune response, and the lipid metabolism pathways) and the findings in recent GWASes. Gene-sets were defined through AmiGO 2 gene-ontology database. Two lists of genes implicated in AD were obtained from the two recent GWASes, Jansen et al.[1] (left) and Kunkle et al.[2] (right), and compared against the three defined gene-sets. The count of overlapping genes between each gene-set and the findings from recent GWASes were shown above. To note, AD-implicated genes were identified through a variety of ways in the GWASes and the overlapping counts in each category were shown.
(DOCX)

S8 Fig Distribution of CADD scores among rare deleterious variants defined by VEP and PolyPhen-2. (DOCX)

S9 Fig Distribution of rare deleterious variants in different mutation categories. (DOCX)

S10 Fig Distribution of pLI scores among endocytic genes. (DOCX)

S11 Fig Overlapping genes between the four gene-sets (endo-system, endosome, lysosome, and trans-Golgi network). (DOCX)

S12 Fig Comparison of age distribution between AD cases and controls in the three datasets (ADSP case-control, AMP-AD case-control, and ADSP Family datasets). (DOCX)

S13 Fig Functional annotation and confirmation of the biological functions of the endo-system gene-set. (EPS)

S1 Table Rare-variant gene-set AD association analysis using PLINK. The OR and P represented the estimated odds ratio and the p-value from the corresponding logistic regression model (or the generalized linear mixed model for family study). P-values were highlighted in red (if <0.05; nominally significant) or green (if <0.00625; gene-set-wide significant). M0 took into account the sequencing location, first ten PCs, total count of rare variants. M1 was M0 plus age and sex. M2 was M1 plus the count of APOE ε2 and ε4 alleles. The P and P* in the meta-analysis across two stages (three datasets) represented the p-values calculated using the fixed-effects inverse variance weighted method by METAL and the Fisher’s method by ‘meta-p,’ respectively. # variants represented the number of rare deleterious variants identified in each dataset for each gene-set. The directions of effects were consistent across nearly all models.
(DOCX)

S2 Table Comparison of stage 2 AMP-AD rare-variant gene-set AD association analysis. The OR and P represented the estimated odds ratio and the p-value from the corresponding logistic regression model (or the generalized linear mixed model for family study). P-values were highlighted in red (if <0.05; nominally significant) or green (if <0.00625; gene-set-wide significant). M0 took into account the sequencing location, first ten PCs, total count of rare variants. M1 was M0 plus age and sex. M2 was M1 plus the count of *APOE* ε2 and ε4 alleles. The stage 2 AMP-AD* cohort represented the largest AMP-AD sub-cohort, ROSMAP.
(DOCX)

S3 Table Rare-variant AD association analysis using the MAGMA burden method. The starred (*) geneset are those excluding the *APOE* gene. The Mu and P-self represented the estimated mean association and the self-contained p-value testing whether an association existed within the tested gene-set. The Beta and P-comp represented the estimated effect size and the competitive p-value testing whether the association within the gene-set was greater than in other genes. P-values were highlighted in red (if <0.05; nominally significant) or green (if <0.00625; gene-set-wide significant). M0 took into account the sequencing location, first ten PCs, total count of rare variants. M1 was M0 plus age and sex. M2 was M1 plus the count of *APOE* ε2 and ε4 alleles. The directions of effects were consistent across nearly all models.
(DOCX)

S4 Table Rare-variant AD association analysis using the MAGMA SNP-wise method. The starred (*) geneset are those excluding the *APOE* gene. The Mu and P-self represented the estimated mean association and the self-contained p-value testing whether an association has existed within the tested gene-set. The Beta and P-comp represented the estimated effect size and the competitive p-value testing whether the association within the gene-set was greater than in other genes. P-values were highlighted in red (if <0.05; nominally significant) or green (if <0.00625; gene-set-wide significant). M0 took into account the sequencing location, first ten PCs, total count of rare variants. M1 was M0 plus age and sex. M2 was M1 plus the count of *APOE* ε2 and ε4 alleles. The directions of effects were consistent across nearly all models.
(DOCX)

S5 Table Rare-variant AD association analysis using PLINK where rare variants were annotated by a combination of VEP, PolyPhen-2, and CADD (>15). The OR and P represented the estimated odds ratio and the p-value from the corresponding logistic regression model (or the generalized linear mixed model for family study). P-values were highlighted in red (if <0.05; nominally significant) or green (if <0.00625; gene-set-wide significant). M0 took into account the sequencing location, first ten PCs, total count of rare variants. M1 was M0 plus age and sex. M2 was M1 plus the count of *APOE* ε2 and ε4 alleles. The stage 2 AMP-AD cohort was analyzed using all sub-cohorts and the largest ROSMAP sub-cohort (71.5% of the total sample size; marked in *). The P and P* in the meta-analysis across two stages (three datasets; AMP-AD* was used here) represented the p-values calculated using the fixed-effects inverse variance weighted method by METAL and the Fisher’s method by ‘meta-p,’ respectively. Similar results could be obtained using the stage 2 AMP-AD. The directions of effects were consistent across nearly all models.
(DOCX)

S6 Table Rare-variant gene-set Braak association analysis using PLINK. The OR and P represented the estimated odds ratio and the p-value from the corresponding logistic regression model (or the generalized linear mixed model for family study). P-values were highlighted in red (if <0.05; nominally significant). M0 took into account the sequencing location, first ten PCs, total count of rare variants. M1 was M0 plus age and sex. M2 was M1 plus the count of *APOE* ε2 and ε4 alleles. The P and P* in the meta-analysis across two stages (two datasets) represented the p-values calculated using the fixed-effects inverse variance weighted method by METAL and the Fisher’s method by ‘meta-p,’ respectively. The directions of effects were consistent across nearly all models.
(DOCX)

S7 Table Rare-variant gene-set AAO and AOD association analysis using PLINK. The OR and P represented the estimated odds ratio and the p-value from the corresponding logistic regression model (or the generalized linear mixed model for family study). P-values were highlighted in red (if <0.05; nominally significant) or green (if <0.00625; gene-set-wide significant). M0 took into account the sequencing location, first ten PCs, total count of rare variants. M1 was M0 plus age and sex. M2 was M1 plus the count of *APOE* ε2 and ε4 alleles. The P and P* in the meta-analysis across two stages (two datasets) represented the p-values calculated using the fixed-effects inverse variance weighted method by METAL and the Fisher’s method by ‘meta-p,’ respectively. The directions of effects were consistent across nearly all models.
(DOCX)

S8 Table Top ten most significant genes in rare-variant single-gene NFT association analysis. The genes were sorted in the descending order of p-values. The meta-analysis was performed using MetaSKAT. P-values below the Bonferroni threshold (α = 4.83*10^−5^; 4.25*10^−5^; 5.17*10^−5^, for ADSP, AMP-AD, and meta-analysis, respectively) were highlighted in red.
(DOCX)

S9 Table Differential expression analysis of three identified genes, *HLA-A*, *SLC26A*, and *ANKRD13D*, between AD cases and controls from the ROSMAP study using six major cell types. Abbreviations: Ex: excitatory neuron; In: inhibitory neuron; Ast: astrocyte; Oli: oligodendrocyte; Opc: oligodendrocyte-precursor-cell; Mic: microglia. Effect: t-statistics calculated using student t-test, representing the direction of effect. P-values are computed using the same method.
(DOCX)

S10 Table Differential expression analysis of three identified genes, *HLA-A*, *SLC26A*, and *ANKRD13D*, between AD cases and control from the ROSMAP study using four cellular subpopulations implicated with AD pathology. Abbreviations: Ex: excitatory neuron; In: inhibitory neuron; Ast: astrocyte; Oli: oligodendrocyte; Opc: oligodendrocyte-precursor-cell; Mic: microglia. Effect: t-statistics calculated using student t-test, representing the direction of effect. P-values are computed using the same method.
(DOCX)

S11 Table Count of singletons and private doubletons within the included rare deleterious variants. The number of total variants represented all rare deleterious variants included under the corresponding MAF threshold.
(DOCX)

S12 Table Rare-variant AD association analysis weighted by pLI scores. The OR and P represented the estimated odds ratio and the p-value from the corresponding logistic regression model (or the generalized linear mixed model for family study). P-values were highlighted in red (if <0.05; nominally significant) or green (if <0.00625; gene-set-wide significant). M0 took into account the sequencing location, first ten PCs, total count of rare variants. M1 was M0 plus age and sex. M2 was M1 plus the count of *APOE* ε2 and ε4 alleles. The P and P* in the meta-analysis across two stages (three datasets) represented the p-values calculated using the fixed-effects inverse variance weighted method by METAL and the Fisher’s method by ‘meta-p,’ respectively.
(DOCX)

S13 Table Rare-variant AD association analysis using gene-sets related to BMI and height. The OR and P represented the estimated odds ratio and the p-value from the corresponding logistic regression model (or the generalized linear mixed model for family study). M0 took into account the sequencing location, first ten PCs, total count of rare variants. M1 was M0 plus age and sex. M2 was M1 plus the count of *APOE* ε2 and ε4 alleles. The P and P* in the meta-analysis across two stages (three datasets) represented the p-values calculated using the fixed-effects inverse variance weighted method by METAL and the Fisher’s method by ‘meta-p,’ respectively.
(DOCX)

S14 Table Number of rare variants passing different HWE cutoffs at different MAF thresholds. Abbreviations: HWE: Hardy-Weinberg Equilibrium; MAF: minor allele frequency.
(DOCX)

S15 Table Top ten most significant genes in rare-variant single-gene AD association analysis. The genes were sorted in the descending order of p-values. The meta-analysis was performed using MetaSKAT. The Bonferroni thresholds were α = 4.18*10^−5^; 4.07*10^−5^; 7.32*10^−5^; 7.79*10^−5^, for ADSP, AMP-AD, ADSP Family, and meta-analysis, respectively.
(DOCX)

S16 Table A full list of the endocytic genes with corresponding coordinates in GRCh38 and the average coverage (DP) in the ADSP and the AMP-AD datasets. (XLSX)
