# Supplementary material for: Rare variants in the endocytic pathway are associated with Alzheimer’s disease, its related phenotypes, and functional consequences
Source: PLoS Genet. 2021 Sep 13;17(9):e1009772. doi: 10.1371/journal.pgen.1009772 (PMC8460036; doi:10.1371/journal.pgen.1009772)
Supplement: S7 Fig — Gene-sets were defined through AmiGO 2 gene-ontology database. Two lists of genes implicated in AD were obtained from the two recent GWASes, Jansen et al.[1] (left) and Kunkle et al.[2] (right), and compared against the three defined gene-sets. The count of overlapping genes between each gene-set and the findings from recent GWASes were shown above. To note, AD-implicated genes were identified through a variety of ways in the GWASes and the overlapping counts in each category were shown. (DOCX) [file pgen.1009772.s007.docx]

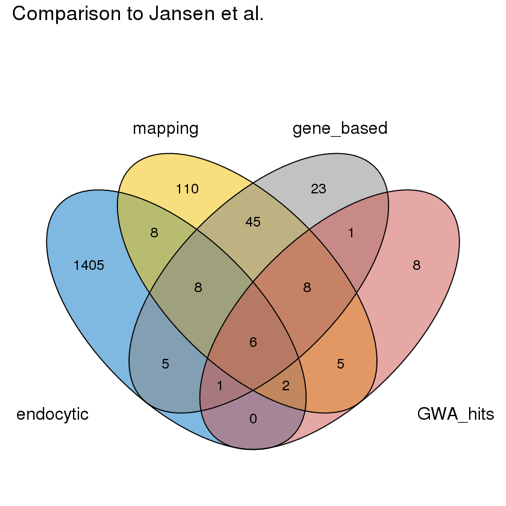

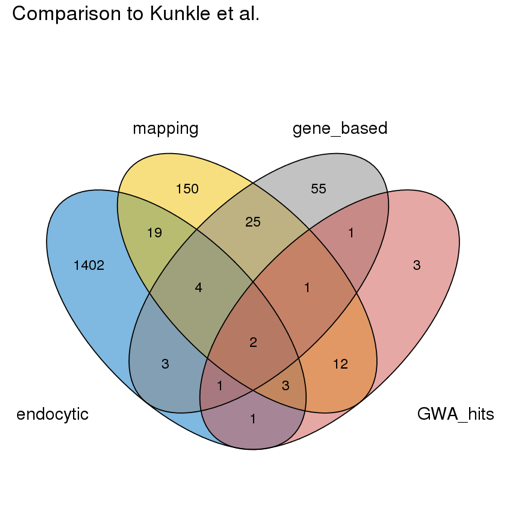


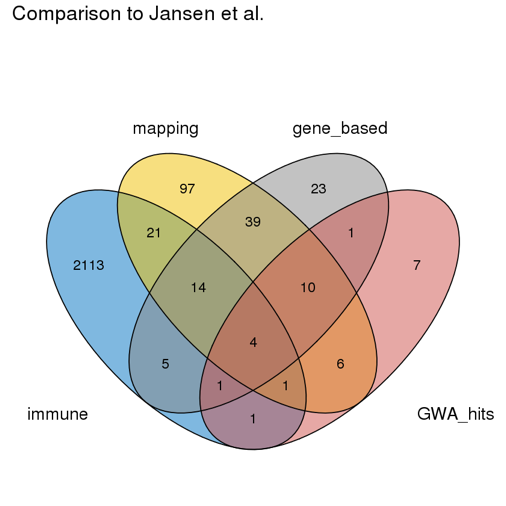

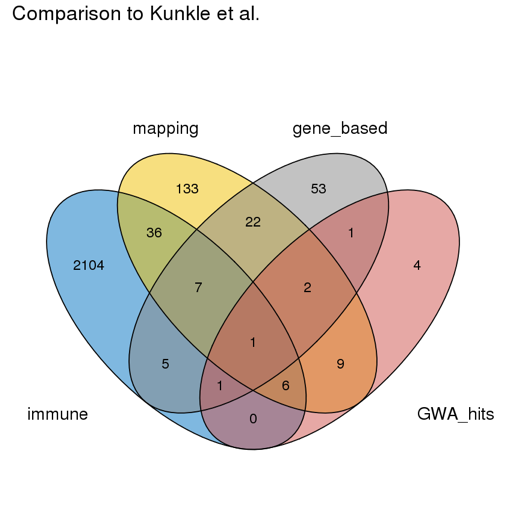


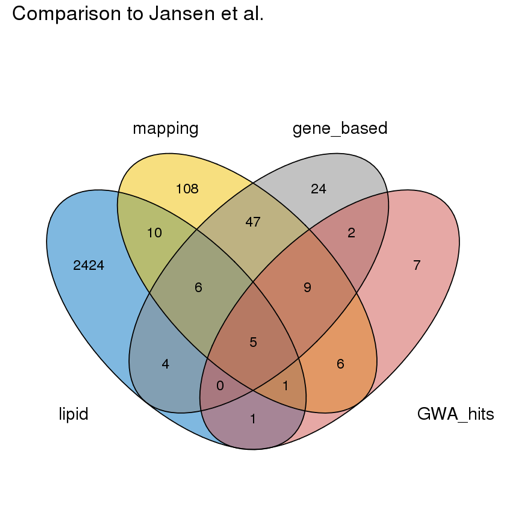

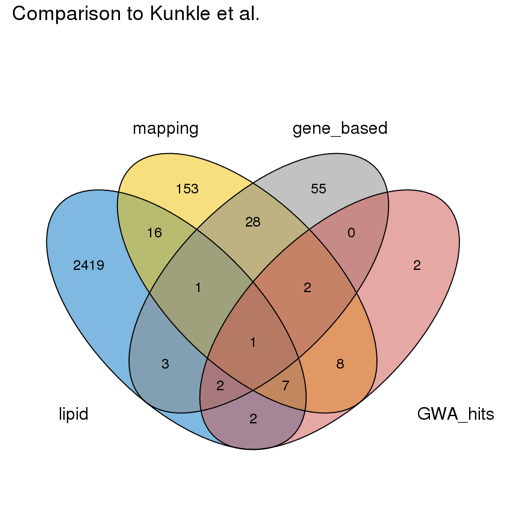


S7 Fig. Overlapping genes between gene-sets (the endocytic, the immune response, and the lipid metabolism pathways) and the findings in recent GWASes.

Gene-sets were defined through AmiGO 2 gene-ontology database. Two lists of genes implicated in AD were obtained from the two recent GWASes, Jansen et al.[1] (left) and Kunkle et al.[2] (right), and compared against the three defined gene-sets. The count of overlapping genes between each gene-set and the findings from recent GWASes were shown above. To note, AD-implicated genes were identified through a variety of ways in the GWASes and the overlapping counts in each category were shown.

Reference

1. Jansen IE, Savage JE, Watanabe K, Bryois J, Williams DM, Steinberg S, et al. Genome-wide meta-analysis identifies new loci and functional pathways influencing Alzheimer's disease risk. Nat Genet. 2019;51(3):404-13.

2. Kunkle BW, Grenier-Boley B, Sims R, Bis JC, Damotte V, Naj AC, et al. Genetic meta-analysis of diagnosed Alzheimer's disease identifies new risk loci and implicates Abeta, tau, immunity and lipid processing. Nat Genet. 2019;51(3):414-30.
