# Supplementary figures and images for: Rare variants in the endocytic pathway are associated with Alzheimer’s disease, its related phenotypes, and functional consequences
Source: PLoS Genet. 2021 Sep 13;17(9):e1009772. doi: 10.1371/journal.pgen.1009772 (PMC8460036; doi:10.1371/journal.pgen.1009772)

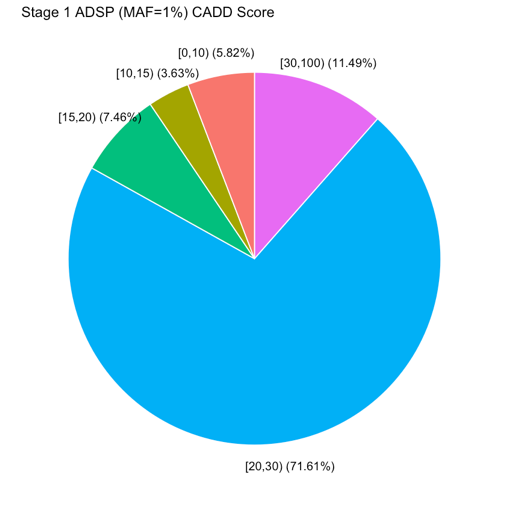

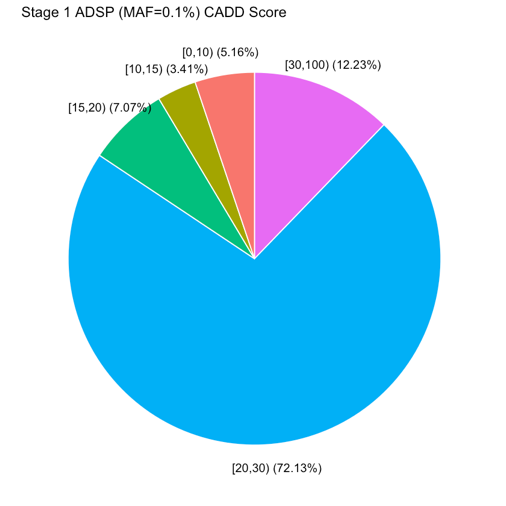


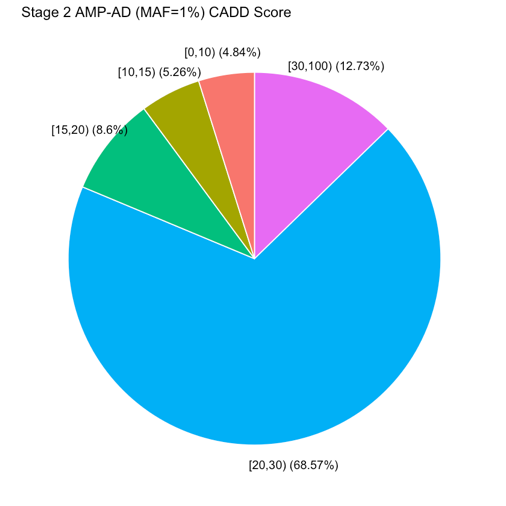

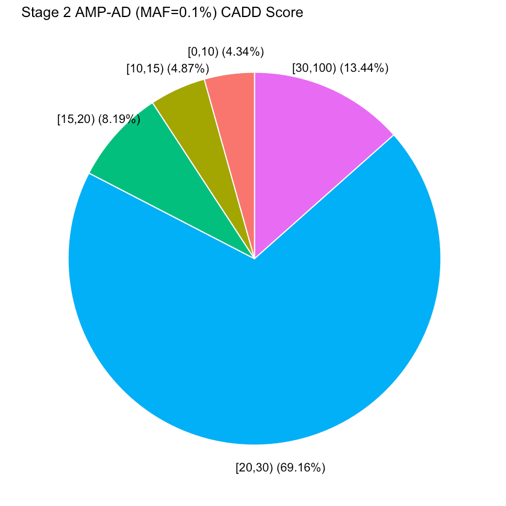


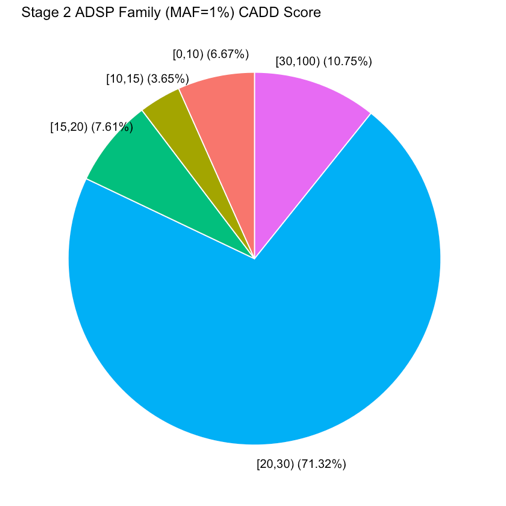

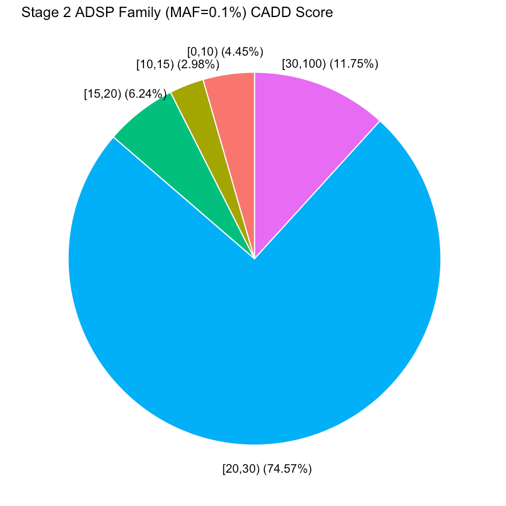


S8 Fig. Distribution of CADD scores among rare deleterious variants defined by VEP and PolyPhen-2.

Supplement: S8 Fig — (DOCX) [file pgen.1009772.s008.docx]

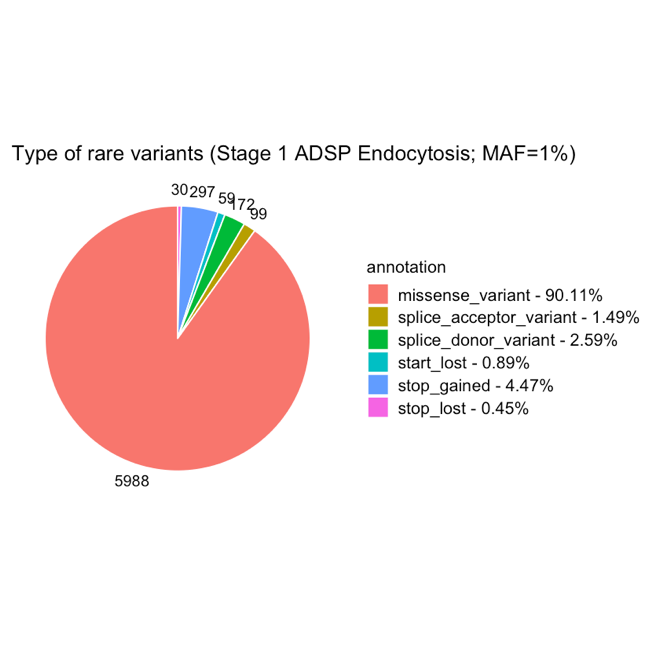

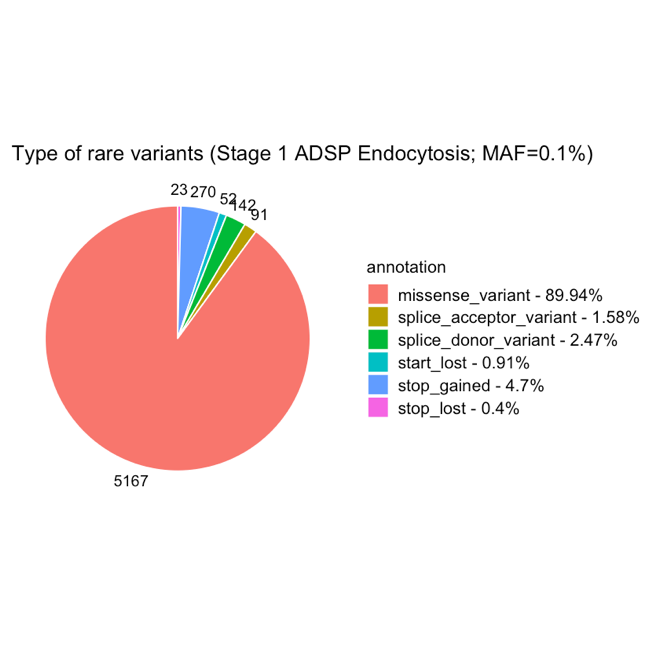


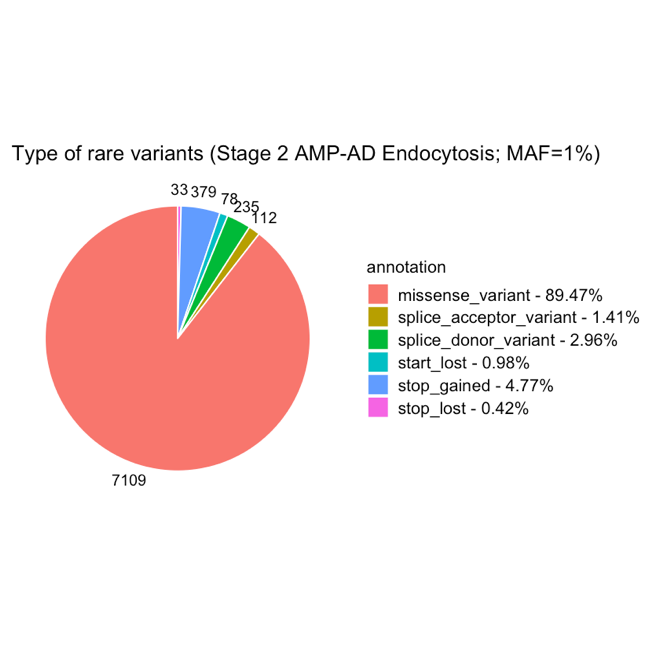

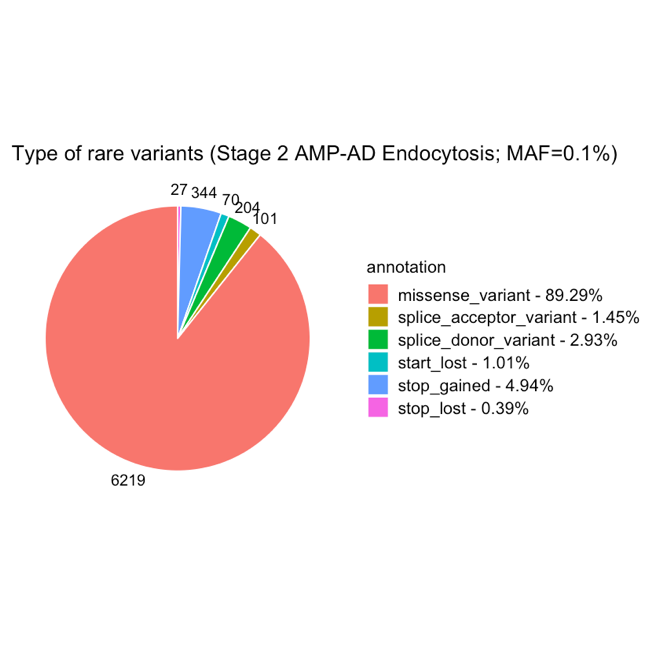


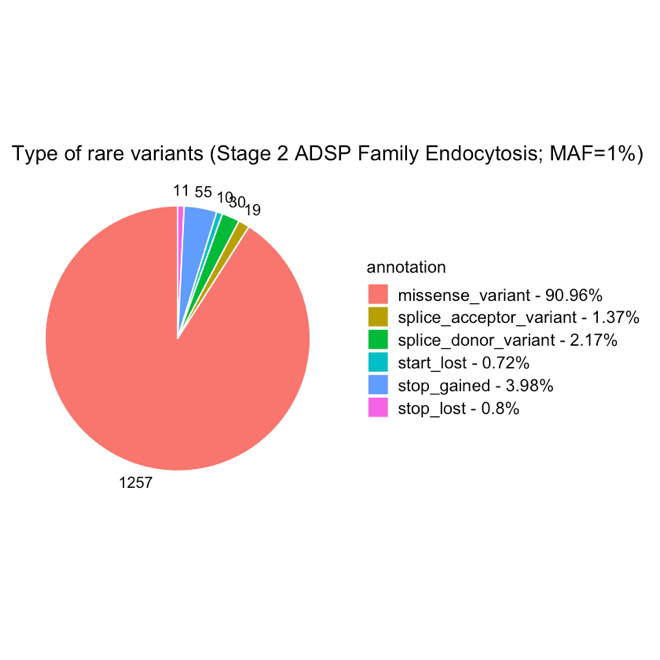

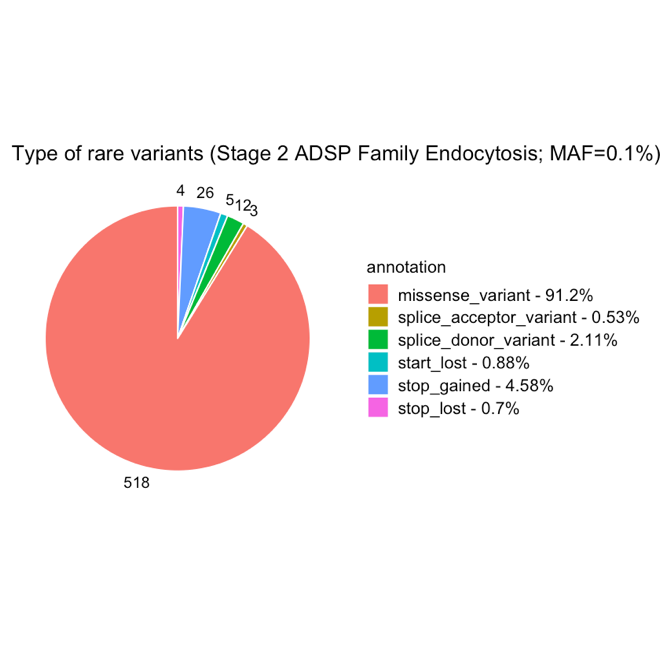


S9 Fig. Distribution of rare deleterious variants in different mutation categories.

Supplement: S9 Fig — (DOCX) [file pgen.1009772.s009.docx]

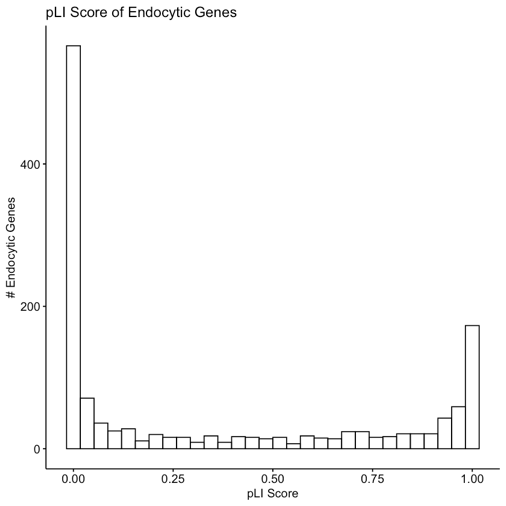


S10 Fig. Distribution of pLI scores among endocytic genes.

Supplement: S10 Fig — (DOCX) [file pgen.1009772.s010.docx]
