# Supplementary material for: Rare variants in the endocytic pathway are associated with Alzheimer’s disease, its related phenotypes, and functional consequences
Source: PLoS Genet. 2021 Sep 13;17(9):e1009772. doi: 10.1371/journal.pgen.1009772 (PMC8460036; doi:10.1371/journal.pgen.1009772)
Supplement: S11 Fig — (DOCX) [file pgen.1009772.s011.docx]

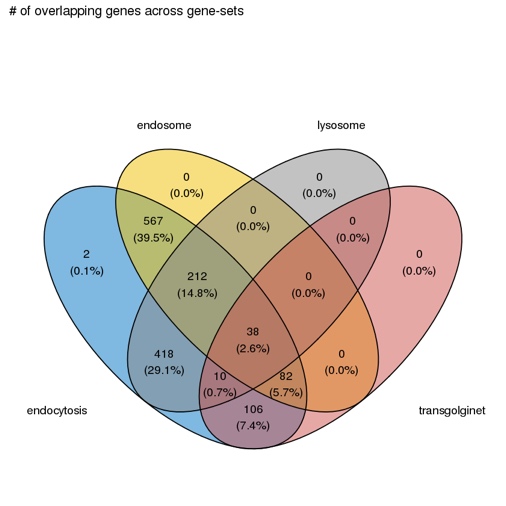


S11 Fig. Overlapping genes between the four gene-sets (endo-system, endosome, lysosome, and trans-Golgi network).
