# Supplementary material for: Rare variants in the endocytic pathway are associated with Alzheimer’s disease, its related phenotypes, and functional consequences
Source: PLoS Genet. 2021 Sep 13;17(9):e1009772. doi: 10.1371/journal.pgen.1009772 (PMC8460036; doi:10.1371/journal.pgen.1009772)
Supplement: S1 Table — The OR and P represented the estimated odds ratio and the p-value from the corresponding logistic regression model (or the generalized linear mixed model for family study). P-values were highlighted in red (if <0.05; nominally significant) or green (if <0.00625; gene-set-wide significant). M0 took into account the sequencing location, first ten PCs, total count of rare variants. M1 was M0 plus age and sex. M2 was M1 plus the count of APOE ε2 and ε4 alleles. The P and P* in the meta-analysis across two stages (three datasets) represented the p-values calculated using the fixed-effects inverse variance weighted method by METAL and the Fisher’s method by ‘meta-p,’ respectively. # variants represented the number of rare deleterious variants identified in each dataset for each gene-set. The directions of effects were consistent across nearly all models. (DOCX) [file pgen.1009772.s014.docx]

| Gene-set | Model | Stage 1 ADSP | | Stage 2 AMP-AD | | Stage 2 ADSP Family | | Meta-analysis | |
| --- | --- | --- | --- | --- | --- | --- | --- | --- | --- |
|  |  | OR | P | OR | P | OR | P | P | P* |
| Endosys | M0 | 1.24 | 1.83E-04 | 1.17 | 5.75E-03 | 1.38 | 1.90E-02 | 3.24E-07 | 5.42E-07 |
|  | M1 | 1.23 | 3.44E-04 | 1.19 | 3.83E-03 | 1.42 | 1.30E-02 | 2.34E-07 | 4.72E-07 |
|  | M2 | 1.20 | 3.07E-03 | 1.17 | 1.30E-02 | 1.35 | 4.20E-02 | 1.58E-05 | 2.83E-05 |
|  | # Variants | 5,745 | | 7,946 | | 1,382 | |  |  |
| Endosome | M0 | 1.17 | 5.56E-03 | 1.08 | 1.82E-01 | 1.41 | 1.00E-02 | 1.14E-04 | 1.35E-04 |
|  | M1 | 1.16 | 8.33E-03 | 1.08 | 1.60E-01 | 1.48 | 4.50E-03 | 7.07E-05 | 8.61E-05 |
|  | M2 | 1.13 | 5.07E-02 | 1.06 | 3.69E-01 | 1.48 | 7.50E-03 | 1.42E-03 | 1.26E-03 |
|  | # Variants | 3,419 | | 4,647 | | 789 | |  |  |
| Lysosome | M0 | 1.10 | 8.99E-02 | 1.15 | 1.05E-02 | 1.19 | 2.00E-01 | 1.39E-03 | 1.61E-03 |
|  | M1 | 1.08 | 1.54E-01 | 1.17 | 6.33E-03 | 1.18 | 2.20E-01 | 1.88E-03 | 1.79E-03 |
|  | M2 | 1.08 | 2.28E-01 | 1.16 | 1.32E-02 | 1.13 | 3.80E-01 | 8.42E-03 | 6.99E-03 |
|  | # Variants | 2,959 | | 4,076 | | 733 | |  |  |
| TransGolgiNet | M0 | 1.15 | 1.94E-02 | 1.09 | 1.06E-01 | 1.04 | 7.80E-01 | 1.46E-02 | 9.19E-03 |
|  | M1 | 1.14 | 2.10E-02 | 1.10 | 8.30E-02 | 1.04 | 7.70E-01 | 1.23E-02 | 7.95E-03 |
|  | M2 | 1.14 | 3.97E-02 | 1.09 | 1.41E-01 | 0.98 | 8.80E-01 | 3.36E-02 | 2.64E-02 |
|  | # Variants | 894 | | 1,215 | | 204 | |  |  |

S1 Table. Rare-variant gene-set AD association analysis using PLINK. The OR and P represented the estimated odds ratio and the p-value from the corresponding logistic regression model (or the generalized linear mixed model for family study). P-values were highlighted in red (if <0.05; nominally significant) or green (if <0.00625; gene-set-wide significant). M0 took into account the sequencing location, first ten PCs, total count of rare variants. M1 was M0 plus age and sex. M2 was M1 plus the count of *APOE* 𝜀2 and 𝜀4 alleles. The P and P* in the meta-analysis across two stages (three datasets) represented the p-values calculated using the fixed-effects inverse variance weighted method by METAL and the Fisher’s method by ‘meta-p,’ respectively. # variants represented the number of rare deleterious variants identified in each dataset for each gene-set. The directions of effects were consistent across nearly all models.
