# Supplementary material for: Rare variants in the endocytic pathway are associated with Alzheimer’s disease, its related phenotypes, and functional consequences
Source: PLoS Genet. 2021 Sep 13;17(9):e1009772. doi: 10.1371/journal.pgen.1009772 (PMC8460036; doi:10.1371/journal.pgen.1009772)
Supplement: S3 Table — The starred (*) geneset are those excluding the APOE gene. The Mu and P-self represented the estimated mean association and the self-contained p-value testing whether an association existed within the tested gene-set. The Beta and P-comp represented the estimated effect size and the competitive p-value testing whether the association within the gene-set was greater than in other genes. P-values were highlighted in red (if <0.05; nominally significant) or green (if <0.00625; gene-set-wide significant). M0 took into account the sequencing location, first ten PCs, total count of rare variants. M1 was M0 plus age and sex. M2 was M1 plus the count of APOE ε2 and ε4 alleles. The directions of effects were consistent across nearly all models. (DOCX) [file pgen.1009772.s016.docx]

| Gene-set | Model | Stage 1 ADSP | | | | Stage 2 AMP-AD | | | | Stage 2 ADSP Family | | | | Meta-analysis | | | |
| --- | --- | --- | --- | --- | --- | --- | --- | --- | --- | --- | --- | --- | --- | --- | --- | --- | --- |
|  |  | Mu | P-self | Beta | P-comp | Mu | P-self | Beta | P-comp | Mu | P-self | Beta | P-comp | MU | P-self | Beta | P-comp |
| Endosys | M0 | 9.06E-02 | 1.02E-03 | 6.53E-02 | 1.30E-02 | 7.68E-02 | 4.88E-03 | 4.08E-02 | 7.41E-02 | 1.15E-01 | 1.35E-03 | 4.49E-02 | 9.28E-02 | 1.55E-01 | 5.16E-08 | 7.98E-02 | 1.90E-03 |
|  | M1 | 9.31E-02 | 7.64E-04 | 5.58E-02 | 2.58E-02 | 7.05E-02 | 8.85E-03 | 3.45E-02 | 1.12E-01 | 1.14E-01 | 1.58E-03 | 4.62E-02 | 8.64E-02 | 1.51E-01 | 1.12E-07 | 7.07E-02 | 4.90E-03 |
|  | M2 | 7.40E-02 | 5.88E-03 | 5.82E-02 | 2.23E-02 | 6.18E-02 | 1.87E-02 | 3.91E-02 | 8.59E-02 | 7.55E-02 | 2.49E-02 | 4.07E-02 | 1.25E-01 | 1.14E-01 | 4.44E-05 | 6.56E-02 | 9.13E-03 |
| Endosome | M0 | 6.59E-02 | 3.94E-02 | 2.92E-02 | 2.15E-01 | 1.22E-01 | 6.11E-04 | 8.31E-02 | 9.66E-03 | 1.71E-01 | 3.22E-04 | 1.10E-01 | 5.78E-03 | 1.91E-01 | 1.13E-07 | 1.09E-01 | 7.33E-04 |
|  | M1 | 8.09E-02 | 1.54E-02 | 3.10E-02 | 1.95E-01 | 1.25E-01 | 4.79E-04 | 8.44E-02 | 9.02E-03 | 1.67E-01 | 4.47E-04 | 1.10E-01 | 5.87E-03 | 1.97E-01 | 4.31E-08 | 1.10E-01 | 6.31E-04 |
|  | M2 | 7.52E-02 | 2.25E-02 | 4.72E-02 | 9.77E-02 | 1.02E-01 | 3.33E-03 | 8.06E-02 | 1.25E-02 | 1.04E-01 | 1.88E-02 | 7.96E-02 | 4.02E-02 | 1.54E-01 | 1.55E-05 | 1.00E-01 | 1.89E-03 |
| Lysosome | M0 | 1.30E-01 | 1.10E-03 | 1.11E-01 | 3.75E-03 | 6.92E-02 | 5.38E-02 | 3.87E-02 | 1.68E-01 | 8.72E-02 | 5.34E-02 | 1.36E-02 | 3.86E-01 | 1.70E-01 | 2.75E-05 | 9.48E-02 | 7.84E-03 |
|  | M1 | 1.18E-01 | 2.73E-03 | 8.91E-02 | 1.40E-02 | 6.87E-02 | 5.51E-02 | 4.09E-02 | 1.55E-01 | 1.17E-01 | 1.53E-02 | 4.38E-02 | 1.75E-01 | 1.71E-01 | 2.36E-05 | 9.40E-02 | 7.88E-03 |
|  | M2 | 1.33E-01 | 9.04E-04 | 1.27E-01 | 9.88E-04 | 7.52E-02 | 4.03E-02 | 5.71E-02 | 8.00E-02 | 9.52E-02 | 3.90E-02 | 5.79E-02 | 1.18E-01 | 1.66E-01 | 3.96E-05 | 1.19E-01 | 1.35E-03 |
| TransGolgiNet | M0 | 1.35E-01 | 3.20E-02 | 9.67E-02 | 8.64E-02 | 1.49E-01 | 2.33E-02 | 1.19E-01 | 4.07E-02 | 2.00E-01 | 1.97E-02 | 8.68E-02 | 1.51E-01 | 2.58E-01 | 1.94E-04 | 1.83E-01 | 3.17E-03 |
|  | M1 | 9.34E-02 | 1.00E-01 | 4.44E-02 | 2.61E-01 | 1.26E-01 | 4.67E-02 | 9.19E-02 | 9.09E-02 | 1.30E-01 | 8.96E-02 | 2.38E-02 | 3.88E-01 | 1.98E-01 | 3.19E-03 | 1.18E-01 | 3.86E-02 |
|  | M2 | 6.35E-02 | 1.92E-01 | 2.82E-02 | 3.43E-01 | 1.05E-01 | 8.05E-02 | 7.84E-02 | 1.29E-01 | 6.79E-02 | 2.42E-01 | -9.06E-03 | 5.41E-01 | 1.28E-01 | 3.96E-02 | 7.46E-02 | 1.35E-01 |
| Endosys* | M0 | 9.15E-02 | 9.25E-04 | 6.63E-02 | 1.20E-02 | 7.71E-02 | 4.77E-03 | 4.11E-02 | 7.29E-02 | 1.14E-01 | 1.57E-03 | 4.27E-02 | 1.04E-01 | 1.55E-01 | 5.13E-08 | 7.98E-02 | 1.90E-03 |
|  | M1 | 9.46E-02 | 6.38E-04 | 5.75E-02 | 2.25E-02 | 7.10E-02 | 8.50E-03 | 3.50E-02 | 1.09E-01 | 1.12E-01 | 1.82E-03 | 4.41E-02 | 9.67E-02 | 1.51E-01 | 1.01E-07 | 7.13E-02 | 4.62E-03 |
|  | M2 | 7.36E-02 | 6.13E-03 | 5.77E-02 | 2.32E-02 | 6.29E-02 | 1.73E-02 | 4.02E-02 | 8.02E-02 | 7.55E-02 | 2.50E-02 | 4.02E-02 | 1.28E-01 | 1.14E-01 | 4.25E-05 | 6.59E-02 | 8.87E-03 |
| Endosome* | M0 | 6.73E-02 | 3.63E-02 | 3.07E-02 | 2.03E-01 | 1.23E-01 | 5.89E-04 | 8.36E-02 | 9.35E-03 | 1.68E-01 | 4.01E-04 | 1.07E-01 | 7.30E-03 | 1.91E-01 | 1.12E-07 | 1.10E-01 | 7.31E-04 |
|  | M1 | 8.35E-02 | 1.31E-02 | 3.36E-02 | 1.76E-01 | 1.26E-01 | 4.46E-04 | 8.52E-02 | 8.50E-03 | 1.64E-01 | 5.50E-04 | 1.07E-01 | 7.32E-03 | 1.98E-01 | 3.77E-08 | 1.11E-01 | 5.75E-04 |
|  | M2 | 7.45E-02 | 2.36E-02 | 4.64E-02 | 1.02E-01 | 1.04E-01 | 2.92E-03 | 8.24E-02 | 1.10E-02 | 1.05E-01 | 1.87E-02 | 7.89E-02 | 4.15E-02 | 1.54E-01 | 1.45E-05 | 1.01E-01 | 1.81E-03 |

S3 Table. Rare-variant AD association analysis using the MAGMA burden method. The starred (*) geneset are those excluding the *APOE* gene. The Mu and P-self represented the estimated mean association and the self-contained p-value testing whether an association existed within the tested gene-set. The Beta and P-comp represented the estimated effect size and the competitive p-value testing whether the association within the gene-set was greater than in other genes. P-values were highlighted in red (if <0.05; nominally significant) or green (if <0.00625; gene-set-wide significant). M0 took into account the sequencing location, first ten PCs, total count of rare variants. M1 was M0 plus age and sex. M2 was M1 plus the count of *APOE* 𝜀2 and 𝜀4 alleles. The directions of effects were consistent across nearly all models.
