# Supplementary material for: Rare variants in the endocytic pathway are associated with Alzheimer’s disease, its related phenotypes, and functional consequences
Source: PLoS Genet. 2021 Sep 13;17(9):e1009772. doi: 10.1371/journal.pgen.1009772 (PMC8460036; doi:10.1371/journal.pgen.1009772)
Supplement: S5 Table — The OR and P represented the estimated odds ratio and the p-value from the corresponding logistic regression model (or the generalized linear mixed model for family study). P-values were highlighted in red (if <0.05; nominally significant) or green (if <0.00625; gene-set-wide significant). M0 took into account the sequencing location, first ten PCs, total count of rare variants. M1 was M0 plus age and sex. M2 was M1 plus the count of APOE ε2 and ε4 alleles. The stage 2 AMP-AD cohort was analyzed using all sub-cohorts and the largest ROSMAP sub-cohort (71.5% of the total sample size; marked in *). The P and P* in the meta-analysis across two stages (three datasets; AMP-AD* was used here) represented the p-values calculated using the fixed-effects inverse variance weighted method by METAL and the Fisher’s method by ‘meta-p,’ respectively. Similar results could be obtained using the stage 2 AMP-AD. The directions of effects were consistent across nearly all models. (DOCX) [file pgen.1009772.s018.docx]

| Gene-set | Model | Stage 1 ADSP | | Stage 2 AMP-AD | | Stage 2 AMP-AD* | | Stage 2 ADSP Family | | Meta-analysis | |
| --- | --- | --- | --- | --- | --- | --- | --- | --- | --- | --- | --- |
|  |  | OR | P | OR | P | OR | P | OR | P | P | P* |
| Endosys | M0 | 1.23 | 2.84E-04 | 1.14 | 1.84E-02 | 1.21 | 7.86E-03 | 1.38 | 1.40E-02 | 4.45E-07 | 8.12E-07 |
|  | M1 | 1.22 | 5.37E-04 | 1.16 | 1.26E-02 | 1.22 | 4.67E-03 | 1.41 | 8.90E-03 | 2.72E-07 | 5.99E-07 |
|  | M2 | 1.19 | 6.60E-03 | 1.16 | 1.41E-02 | 1.24 | 3.93E-03 | 1.35 | 1.90E-02 | 4.48E-06 | 9.56E-06 |
| Endosome | M0 | 1.18 | 4.38E-03 | 1.07 | 2.13E-01 | 1.16 | 4.13E-02 | 1.46 | 2.90E-03 | 5.56E-06 | 1.01E-05 |
|  | M1 | 1.17 | 6.96E-03 | 1.08 | 1.79E-01 | 1.17 | 3.09E-02 | 1.54 | 8.70E-04 | 2.29E-06 | 4.05E-06 |
|  | M2 | 1.12 | 7.01E-02 | 1.08 | 2.16E-01 | 1.16 | 4.71E-02 | 1.55 | 1.10E-03 | 4.58E-05 | 5.56E-05 |
| Lysosome | M0 | 1.09 | 1.33E-01 | 1.12 | 3.46E-02 | 1.19 | 1.09E-02 | 1.21 | 1.40E-01 | 1.43E-03 | 1.71E-03 |
|  | M1 | 1.07 | 2.17E-01 | 1.14 | 2.17E-02 | 1.22 | 4.19E-03 | 1.21 | 1.40E-01 | 1.29E-03 | 1.16E-03 |
|  | M2 | 1.06 | 3.42E-01 | 1.14 | 2.85E-02 | 1.22 | 6.57E-03 | 1.16 | 2.20E-01 | 4.72E-03 | 3.56E-03 |
| TransGolgiNet | M0 | 1.16 | 1.00E-02 | 1.09 | 1.01E-01 | 1.08 | 2.27E-01 | 1.08 | 5.20E-01 | 1.06E-02 | 7.22E-03 |
|  | M1 | 1.16 | 1.17E-02 | 1.10 | 6.94E-02 | 1.08 | 2.18E-01 | 1.08 | 5.50E-01 | 1.21E-02 | 8.28E-03 |
|  | M2 | 1.15 | 2.69E-02 | 1.10 | 1.04E-01 | 1.09 | 2.17E-01 | 0.99 | 9.30E-01 | 4.12E-02 | 2.63E-02 |

S5 Table. Rare-variant AD association analysis using PLINK where rare variants were annotated by a combination of VEP, PolyPhen-2, and CADD (>15). The OR and P represented the estimated odds ratio and the p-value from the corresponding logistic regression model (or the generalized linear mixed model for family study). P-values were highlighted in red (if <0.05; nominally significant) or green (if <0.00625; gene-set-wide significant). M0 took into account the sequencing location, first ten PCs, total count of rare variants. M1 was M0 plus age and sex. M2 was M1 plus the count of *APOE* 𝜀2 and 𝜀4 alleles. The stage 2 AMP-AD cohort was analyzed using all sub-cohorts and the largest ROSMAP sub-cohort (71.5% of the total sample size; marked in *). The P and P* in the meta-analysis across two stages (three datasets; AMP-AD* was used here) represented the p-values calculated using the fixed-effects inverse variance weighted method by METAL and the Fisher’s method by ‘meta-p,’ respectively. Similar results could be obtained using the stage 2 AMP-AD. The directions of effects were consistent across nearly all models.
