# Supplementary material for: Rare variants in the endocytic pathway are associated with Alzheimer’s disease, its related phenotypes, and functional consequences
Source: PLoS Genet. 2021 Sep 13;17(9):e1009772. doi: 10.1371/journal.pgen.1009772 (PMC8460036; doi:10.1371/journal.pgen.1009772)
Supplement: S8 Table — The genes were sorted in the descending order of p-values. The meta-analysis was performed using MetaSKAT. P-values below the Bonferroni threshold (α = 4.83*10−5; 4.25*10−5; 5.17*10−5, for ADSP, AMP-AD, and meta-analysis, respectively) were highlighted in red. (DOCX) [file pgen.1009772.s021.docx]

| Stage 1 ADSP | | Stage 2 AMP-AD | | Meta-analysis | |
| --- | --- | --- | --- | --- | --- |
| Gene | P | Gene | P | Gene | P |
| CD300LG | 1.41E-06 | HAVCR2 | 4.42E-04 | ANKRD13D | 3.56E-05 |
| ANKRD13D | 1.46E-06 | LNPEP | 1.12E-03 | IL1B | 5.34E-05 |
| TJAP1 | 5.80E-06 | ANKRD13A | 1.56E-03 | DYNC1H1 | 2.17E-03 |
| PLBD1 | 9.16E-06 | PCSK7 | 1.86E-03 | HPS4 | 3.68E-03 |
| IL1B | 1.73E-05 | CPNE1 | 2.28E-03 | TLR3 | 4.15E-03 |
| LLGL1 | 1.79E-05 | TLR9 | 2.40E-03 | KREMEN2 | 5.00E-03 |
| HPS4 | 9.27E-05 | AP2A2 | 2.58E-03 | HAVCR2 | 5.30E-03 |
| STAMBP | 1.19E-04 | OMD | 2.96E-03 | ARFGEF2 | 5.78E-03 |
| LRRK2 | 3.58E-04 | RAB17 | 3.87E-03 | PLEKHA8 | 5.85E-03 |
| DBNL | 1.12E-03 | ATP6V0A2 | 4.20E-03 | EZR | 6.76E-03 |

S8 Table. Top ten most significant genes in rare-variant single-gene NFT association analysis. The genes were sorted in the descending order of p-values. The meta-analysis was performed using MetaSKAT. P-values below the Bonferroni threshold (𝛼=4.83*10^-5^; 4.25*10^-5^; 5.17*10^-5^, for ADSP, AMP-AD, and meta-analysis, respectively) were highlighted in red.
