# Supplementary material for: Rare variants in the endocytic pathway are associated with Alzheimer’s disease, its related phenotypes, and functional consequences
Source: PLoS Genet. 2021 Sep 13;17(9):e1009772. doi: 10.1371/journal.pgen.1009772 (PMC8460036; doi:10.1371/journal.pgen.1009772)
Supplement: S9 Table — Abbreviations: Ex: excitatory neuron; In: inhibitory neuron; Ast: astrocyte; Oli: oligodendrocyte; Opc: oligodendrocyte-precursor-cell; Mic: microglia. Effect: t-statistics calculated using student t-test, representing the direction of effect. P-values are computed using the same method. (DOCX) [file pgen.1009772.s022.docx]

| Gene name | Cell type | Effect | P-value |
| --- | --- | --- | --- |
| *ANKRD13D* | Ast | 2.57 | 1.07E-02 |
|  | Ex | 8.79 | 1.92E-18 |
|  | In | 2.19 | 2.84E-02 |
|  | Mic | -1.75 | 8.26E-02 |
|  | Oli | -3.14 | 1.78E-03 |
|  | Opc | 0.82 | 4.14E-01 |
| *HLA-A* | Ast | -1.09 | 2.76E-01 |
|  | Ex | -0.03 | 9.79E-01 |
|  | In | -4.45 | 9.72E-06 |
|  | Mic | -2.98 | 3.07E-03 |
|  | Oli | -1.28 | 2.01E-01 |
|  | Opc | -1.46 | 1.45E-01 |
| *SLC26A7* | Ast | -1.76 | 9.12E-02 |
|  | Ex | 2.88 | 4.85E-03 |
|  | In | 0.50 | 6.28E-01 |
|  | Mic | 1.22 | 2.48E-01 |
|  | Oli | -0.40 | 6.90E-01 |
|  | Opc | 0.12 | 9.16E-01 |

S9 Table. Differential expression analysis of three identified genes, *HLA-A*, *SLC26A*, and *ANKRD13D*, between AD cases and controls from the ROSMAP study using six major cell types.

Abbreviations: Ex: excitatory neuron; In: inhibitory neuron; Ast: astrocyte; Oli: oligodendrocyte; Opc: oligodendrocyte-precursor-cell; Mic: microglia. Effect: t-statistics calculated using student t-test, representing the direction of effect. P-values are computed using the same method.
