# Supplementary material for: Rare variants in the endocytic pathway are associated with Alzheimer’s disease, its related phenotypes, and functional consequences
Source: PLoS Genet. 2021 Sep 13;17(9):e1009772. doi: 10.1371/journal.pgen.1009772 (PMC8460036; doi:10.1371/journal.pgen.1009772)
Supplement: S10 Table — Abbreviations: Ex: excitatory neuron; In: inhibitory neuron; Ast: astrocyte; Oli: oligodendrocyte; Opc: oligodendrocyte-precursor-cell; Mic: microglia. Effect: t-statistics calculated using student t-test, representing the direction of effect. P-values are computed using the same method. (DOCX) [file pgen.1009772.s023.docx]

| Gene name | Cell type | Effect | P value |
| --- | --- | --- | --- |
| ANKRD13D | Ex4 | 5.49 | 5.76E-08 |
|  | In0 | 2.11 | 3.59E-02 |
|  | Ast1 | 1.37 | 1.78E-01 |
|  | Oli0 | -1.59 | 1.12E-01 |
| HLA-A | Ex4 | 0.53 | 5.99E-01 |
|  | In0 | -1.59 | 1.14E-01 |
|  | Ast1 | 0.86 | 3.91E-01 |
|  | Oli0 | -1.36 | 1.76E-01 |
| SLC26A7 | Ex4 | 2.11 | 8.99E-02 |
|  | In0 | 0.93 | 4.37E-01 |
|  | Ast1 | -2.24 | 2.06E-01 |
|  | Oli0 | -0.26 | 7.97E-01 |

S10 Table. Differential expression analysis of three identified genes, *HLA-A*, *SLC26A*, and *ANKRD13D*, between AD cases and control from the ROSMAP study using four cellular subpopulations implicated with AD pathology

Abbreviations: Ex: excitatory neuron; In: inhibitory neuron; Ast: astrocyte; Oli: oligodendrocyte; Opc: oligodendrocyte-precursor-cell; Mic: microglia. Effect: t-statistics calculated using student t-test, representing the direction of effect. P-values are computed using the same method.
