# Supplementary material for: Rare variants in the endocytic pathway are associated with Alzheimer’s disease, its related phenotypes, and functional consequences
Source: PLoS Genet. 2021 Sep 13;17(9):e1009772. doi: 10.1371/journal.pgen.1009772 (PMC8460036; doi:10.1371/journal.pgen.1009772)
Supplement: S11 Table — The number of total variants represented all rare deleterious variants included under the corresponding MAF threshold. (DOCX) [file pgen.1009772.s024.docx]

|  | Stage 1 ADSP | | Stage 2 AMP-AD | | Stage 2 ADSP Family | |
| --- | --- | --- | --- | --- | --- | --- |
| Endocytic gene-set | MAF 1% | MAF 0.1% | MAF 1% | MAF 0.1% | MAF 1% | MAF 0.1% |
| # of Singletons | 5,113 | 5,011 | 5,878 | 5,803 | 618 | 305 |
| # of Total variants | 6,645 | 5,745 | 7,946 | 6,965 | 1,382 | 568 |
| # of Private Doubletons | 0 | 0 | 2 | 2 | 0 | 0 |
| Percentage of Singletons | 76.9% | 87.2% | 74.0% | 83.3% | 44.7% | 53.7% |

S11 Table. Count of singletons and private doubletons within the included rare deleterious variants. The number of total variants represented all rare deleterious variants included under the corresponding MAF threshold.
