# Supplementary material for: Rare variants in the endocytic pathway are associated with Alzheimer’s disease, its related phenotypes, and functional consequences
Source: PLoS Genet. 2021 Sep 13;17(9):e1009772. doi: 10.1371/journal.pgen.1009772 (PMC8460036; doi:10.1371/journal.pgen.1009772)
Supplement: S12 Table — The OR and P represented the estimated odds ratio and the p-value from the corresponding logistic regression model (or the generalized linear mixed model for family study). P-values were highlighted in red (if <0.05; nominally significant) or green (if <0.00625; gene-set-wide significant). M0 took into account the sequencing location, first ten PCs, total count of rare variants. M1 was M0 plus age and sex. M2 was M1 plus the count of APOE ε2 and ε4 alleles. The P and P* in the meta-analysis across two stages (three datasets) represented the p-values calculated using the fixed-effects inverse variance weighted method by METAL and the Fisher’s method by ‘meta-p,’ respectively. (DOCX) [file pgen.1009772.s025.docx]

| Gene-set | Model | Stage 1 ADSP | | Stage 2 AMP-AD | | Stage 2 ADPS Family | | Meta-analysis | |
| --- | --- | --- | --- | --- | --- | --- | --- | --- | --- |
|  |  | OR | OR | OR | P | OR | P | P | P* |
| Endosys | M0 | 1.17 | 5.82E-03 | 1.11 | 1.50E-01 | 1.12 | 3.80E-01 | 3.38E-03 | 2.57E-03 |
|  | M1 | 1.17 | 6.59E-03 | 1.11 | 1.31E-01 | 1.15 | 2.60E-01 | 1.99E-03 | 1.86E-03 |
|  | M2 | 1.16 | 2.47E-02 | 1.12 | 1.16E-01 | 1.13 | 3.60E-01 | 6.26E-03 | 6.44E-03 |
| Endosome | M0 | 1.12 | 5.18E-02 | 1.08 | 2.69E-01 | 1.02 | 9.00E-01 | 6.67E-02 | 4.44E-02 |
|  | M1 | 1.11 | 6.41E-02 | 1.09 | 2.27E-01 | 1.04 | 7.20E-01 | 4.84E-02 | 3.88E-02 |
|  | M2 | 1.08 | 2.03E-01 | 1.09 | 2.46E-01 | 1.05 | 6.70E-01 | 9.86E-02 | 8.97E-02 |
| Lysosome | M0 | 1.07 | 2.80E-01 | 1.16 | 3.07E-02 | 1.19 | 1.70E-01 | 7.73E-03 | 8.52E-03 |
|  | M1 | 1.06 | 3.55E-01 | 1.18 | 1.87E-02 | 1.22 | 1.20E-01 | 5.29E-03 | 5.25E-03 |
|  | M2 | 1.04 | 5.92E-01 | 1.19 | 1.95E-02 | 1.19 | 1.90E-01 | 1.57E-02 | 1.17E-02 |
| TransGolgiNet | M0 | 1.04 | 4.78E-01 | 0.99 | 8.93E-01 | 1.08 | 5.40E-01 | 4.00E-01 | 3.53E-01 |
|  | M1 | 1.05 | 4.48E-01 | 0.99 | 8.63E-01 | 1.08 | 5.20E-01 | 3.63E-01 | 3.38E-01 |
|  | M2 | 1.09 | 1.77E-01 | 0.98 | 8.05E-01 | 1.07 | 6.00E-01 | 2.20E-01 | 2.18E-01 |

S12 Table. Rare-variant AD association analysis weighted by pLI scores. The OR and P represented the estimated odds ratio and the p-value from the corresponding logistic regression model (or the generalized linear mixed model for family study). P-values were highlighted in red (if <0.05; nominally significant) or green (if <0.00625; gene-set-wide significant). M0 took into account the sequencing location, first ten PCs, total count of rare variants. M1 was M0 plus age and sex. M2 was M1 plus the count of *APOE* 𝜀2 and 𝜀4 alleles. The P and P* in the meta-analysis across two stages (three datasets) represented the p-values calculated using the fixed-effects inverse variance weighted method by METAL and the Fisher’s method by ‘meta-p,’ respectively.
