# Supplementary material for: Rare variants in the endocytic pathway are associated with Alzheimer’s disease, its related phenotypes, and functional consequences
Source: PLoS Genet. 2021 Sep 13;17(9):e1009772. doi: 10.1371/journal.pgen.1009772 (PMC8460036; doi:10.1371/journal.pgen.1009772)
Supplement: S14 Table — Abbreviations: HWE: Hardy-Weinberg Equilibrium; MAF: minor allele frequency. (DOCX) [file pgen.1009772.s027.docx]

|  | Stage 1 ADSP | | Stage 2 AMP-AD | | Stage 2 ADSP Family | |
| --- | --- | --- | --- | --- | --- | --- |
| HWE cutoff | MAF 1% | MAF 0.1% | MAF 1% | MAF 0.1% | MAF 1% | MAF 0.1% |
| Cutoff at 0.001 | 24,775,258 | 17,718,944 | 31,871,709 | 23,352,094 | 6,308,504 | 48,660 |
| Cutoff at 5e-8 | 24,779,990 | 17,719,782 | 31,896,945 | 23,366,281 | 6,308,811 | 48,661 |
| Percentage gained | 0.019% | 0.0047% | 0.079% | 0.061% | 0.0049% | 0.0021% |

S14 Table. Number of rare variants passing different HWE cutoffs at different MAF thresholds.

Abbreviations: HWE: Hardy-Weinberg Equilibrium; MAF: minor allele frequency.
