# Supplementary material for: Rare variants in the endocytic pathway are associated with Alzheimer’s disease, its related phenotypes, and functional consequences
Source: PLoS Genet. 2021 Sep 13;17(9):e1009772. doi: 10.1371/journal.pgen.1009772 (PMC8460036; doi:10.1371/journal.pgen.1009772)
Supplement: S15 Table — The genes were sorted in the descending order of p-values. The meta-analysis was performed using MetaSKAT. The Bonferroni thresholds were α = 4.18*10−5; 4.07*10−5; 7.32*10−5; 7.79*10−5, for ADSP, AMP-AD, ADSP Family, and meta-analysis, respectively. (DOCX) [file pgen.1009772.s028.docx]

| Stage 1 ADSP | | Stage 2 AMP-AD | | Stage 2 ADSP Family | | Meta-analysis | |
| --- | --- | --- | --- | --- | --- | --- | --- |
| Gene | P | Gene | P | Gene | P | Gene | P |
| SLC17A3 | 2.38E-03 | ZFYVE16 | 1.18E-03 | SPNS1 | 3.36E-03 | SORL1 | 2.02E-03 |
| DDIT3 | 4.20E-03 | MFSD8 | 2.00E-03 | MPO | 5.12E-03 | ATP6V0A2 | 3.27E-03 |
| SERPINB13 | 6.15E-03 | PDIA3 | 2.82E-03 | PRSS16 | 5.77E-03 | PRSS16 | 5.47E-03 |
| DAGLB | 6.60E-03 | CLCN7 | 3.82E-03 | FLT1 | 6.15E-03 | ATP6V1A | 7.39E-03 |
| SDC4 | 7.88E-03 | EGFR | 4.65E-03 | TYRP1 | 1.33E-02 | TMEM108 | 9.41E-03 |
| VASN | 8.39E-03 | ABCB9 | 4.77E-03 | ZFYVE9 | 1.39E-02 | GPR137B | 9.69E-03 |
| VAC14 | 9.69E-03 | ABCB9 | 4.77E-03 | RAB27B | 1.52E-02 | CHST4 | 1.06E-02 |
| HRNR | 1.08E-02 | CHMP2B | 5.66E-03 | AP5M1 | 1.78E-02 | SNX17 | 1.33E-02 |
| UNC93B1 | 1.18E-02 | ATP6V1C2 | 7.51E-03 | CDIP1 | 1.89E-02 | AGRN | 1.67E-02 |
| TMEM127 | 1.29E-02 | ATP8A2 | 7.83E-03 | WDR48 | 2.26E-02 | ARRDC3 | 1.90E-02 |

S15 Table. Top ten most significant genes in rare-variant single-gene AD association analysis. The genes were sorted in the descending order of p-values. The meta-analysis was performed using MetaSKAT. The Bonferroni thresholds were 𝛼=4.18*10^-5^; 4.07*10^-5^; 7.32*10^-5^; 7.79*10^-5^, for ADSP, AMP-AD, ADSP Family, and meta-analysis, respectively.
